# Extended Condensed Ultraphosphate Frameworks with Monovalent Ions Combine Lithium Mobility with High Computed Electrochemical Stability

**DOI:** 10.1021/jacs.1c07874

**Published:** 2021-10-22

**Authors:** Guopeng Han, Andrij Vasylenko, Alex R. Neale, Benjamin B. Duff, Ruiyong Chen, Matthew S. Dyer, Yun Dang, Luke M. Daniels, Marco Zanella, Craig M. Robertson, Laurence J. Kershaw Cook, Anna-Lena Hansen, Michael Knapp, Laurence J. Hardwick, Frédéric Blanc, John B. Claridge, Matthew J. Rosseinsky

**Affiliations:** † Department of Chemistry, University of Liverpool, Crown Street, Liverpool, L69 7ZD, United Kingdom; ‡ Stephenson Institute for Renewable Energy, University of Liverpool, Peach Street, Liverpool L69 7ZF, United Kingdom; § Institute for Applied Materials - Energy Storage Systems, Karlsruhe Institute of Technology, Hermann-von-Helmholtz-Platz 1, 76344 Eggenstein-Leopoldshafen, Germany

## Abstract

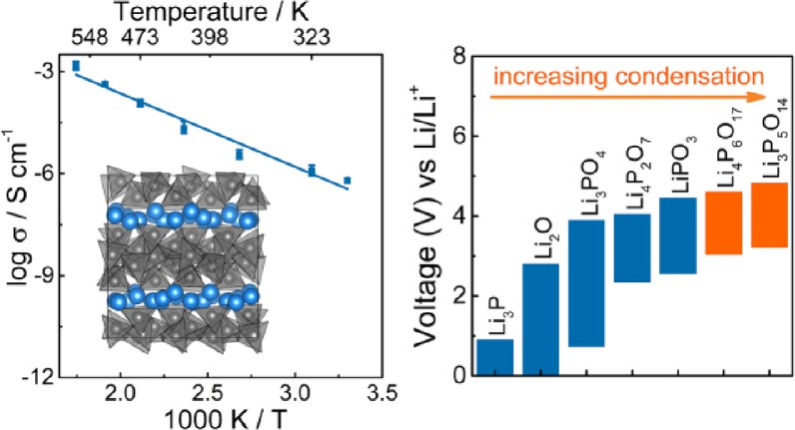

Extended anionic frameworks based on condensation of polyhedral main group non-metal anions offer a wide range of structure types. Despite the widespread chemistry and earth abundance of phosphates and silicates, there are no reports of extended ultraphosphate anions with lithium. We describe the lithium ultraphosphates Li_3_P_5_O_14_ and Li_4_P_6_O_17_ based on extended layers and chains of phosphate, respectively. Li_3_P_5_O_14_ presents a complex structure containing infinite ultraphosphate layers with 12-membered rings that are stacked alternately with lithium polyhedral layers. Two distinct vacant tetrahedral sites were identified at the end of two distinct finite Li_6_O_16_^26–^ chains. Li_4_P_6_O_17_ features a new type of loop-branched chain defined by six PO_4_^3–^ tetrahedra. The ionic conductivities and electrochemical properties of Li_3_P_5_O_14_ were examined by impedance spectroscopy combined with DC polarization, NMR spectroscopy, and galvanostatic plating/stripping measurements. The structure of Li_3_P_5_O_14_ enables three-dimensional lithium migration that affords the highest ionic conductivity (8.5(5) × 10^–7^ S cm^–1^ at room temperature for bulk), comparable to that of commercialized LiPON glass thin film electrolytes, and lowest activation energy (0.43(7) eV) among all reported ternary Li–P–O phases. Both new lithium ultraphosphates are predicted to have high thermodynamic stability against oxidation, especially Li_3_P_5_O_14_, which is predicted to be stable to 4.8 V, significantly higher than that of LiPON and other solid electrolytes. The condensed phosphate units defining these ultraphosphate structures offer a new route to optimize the interplay of conductivity and electrochemical stability required, for example, in cathode coatings for lithium ion batteries.

## 1 Introduction

**Figure 1:**
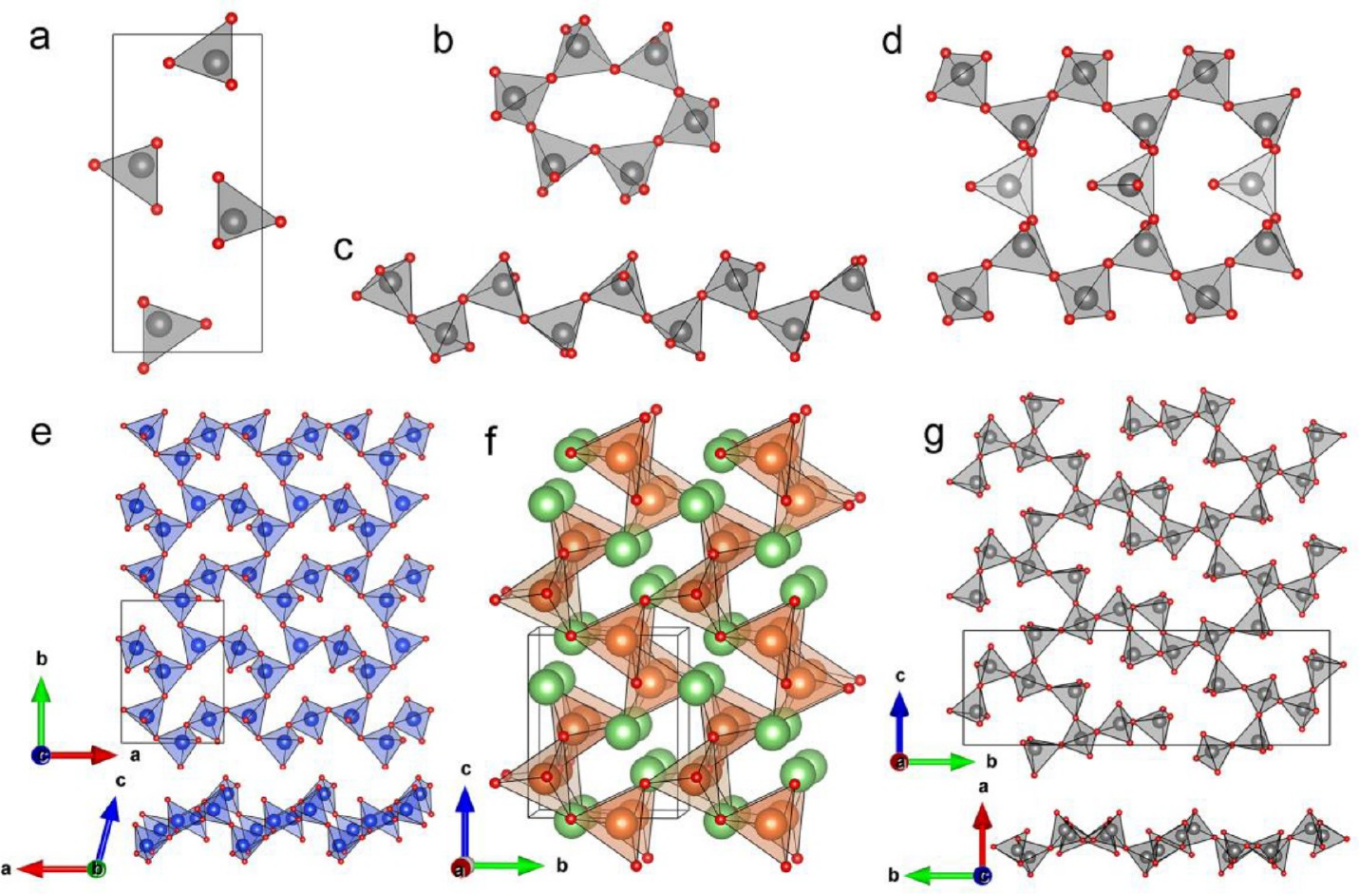
Arrangement of PO_4_^3–^ tetrahedra in four types of phosphates, showing (a) the isolated PO_4_^3–^ tetrahedra in orthophosphate Li_3_PO_4_, (b) 0D 2-connected single P_6_O_18_^6–^ ring in the cyclophosphate Li_6_P_6_O_18_ and (c) 1D 2-connected single PO_3_^–^ ribbon in the polyphosphate LiPO_3_, and (d) 1D 2,3-connected double P_5_O_14_^3–^ ribbon in orthorhombic HoP_5_O_14_ (ultraphosphate); (e) 2D 2,3-connected layer with 8-membered rings in Na_2_ZnSi_3_O_8_; (f) 3D 4-connected AlO_2_^–^ framework with 6-membered channels in γ-LiAlO_2_; (g) 2D 2,3-connected P_4_O_11_^2–^ layer with a combination of 4- and 16-membered rings in ZnP_4_O_11_. Li, P, Al, Si, and O atoms are shown in green, gray, orange, blue, and red, respectively. AlO_4_^5–^ tetrahedra orange, SiO_4_^4–^ tetrahedra blue, PO_4_^3–^ tetrahedra gray.

Phosphates consisting of PO_4_^3–^ tetrahedra can adopt isolated, linear, cyclic, and branched anionic substructures, giving rise to orthophosphate, polyphosphate, cyclophosphate, and ultraphosphate structures, respectively.^1,2^ In the first three of these families, PO_4_^3–^ tetrahedra share zero (isolated tetrahedra), one (terminal tetrahedra), or two (internal tetrahedra) of their oxygens with neighboring tetrahedra, resulting in unbranched 0-, 1-, 2-, or mixed 1,2-connected anions, so only unbranched zero-dimensional (0D) or 1D anions are available, e.g., 0D unbranched 2-connected single P_6_O_18_^6–^ rings in the cyclophosphate Li_6_P_6_O_18_ and 1D 2-connected PO_3_^–^ chains in the polyphosphate LiPO_3_, as shown in Figure 1a–1c.^3,4^ In contrast, ultraphosphates present branched anions that are generated by combining internal tetrahedra with branching PO_4_^3–^ tetrahedra that share three of their oxygens with other tetrahedra (Figure 1d).^5^ This produces richer structural chemistry arising from topologically nonlinear linking, which generates 2,3-connected nets that lie between purely 3-connected phosphoric anhydride P_2_O_5_ and the 2-connected cyclophosphates. The arising anion geometries are more diverse than in other types of phosphates; e.g., ultraphosphates could adopt 0D (finite P_8_O_23_^6–^ groups in Na_3_FeP_8_O_23_),^6^ 1D (infinite P_5_O_14_^3–^ ribbons in orthorhombic HoP_5_O_14_),^5^ 2D (infinite P_4_O_11_^2–^ layers in CaP_4_O_11_),^7,8^ or even 3D (infinite P_6_O_17_^4–^ frameworks in (UO_2_)_2_P_6_O_17_)^9^ anionic geometries.

Ultraphosphates have been shown to be attractive matrices for lasing materials due to their desirable optical properties, which are strongly dependent upon their unique crystal structural features.^10−12^ Thus far, a series of divalent, trivalent, or mixed cation ultraphosphates were reported, such as MgP_4_O_11_,^13^ YP_5_O_14_,^14,15^ and Na_3_MP_8_O_23_ (M = Fe, Al, Ga).^6,16^ The structural chemistry of ultraphosphates is somewhat analogous to that of silicates and aluminates. Crystal structures with 2,3-connected anionic networks occur in polysilicates, phyllosilicates, and polyaluminates, such as loop-branched single chain Si_4_O_11_^4–^ in Li_2_Mg_2_Si_4_O_11_,^17^ double chain Al_3_O_8_^7–^ in Na_7_Al_3_O_8_,^18,19^ and infinite single layer Si_3_O_8_^4–^ in Na_2_ZnSi_3_O_8_ (Figure 1e).^20^ One major difference between ultraphosphates and silicates or aluminates is the connectedness of the tetrahedra. The PO_4_^3–^ tetrahedra can share a maximum of three corners in ultraphosphates, limiting their connectivity to three, while SiO_4_^4–^ and AlO_4_^5–^ tetrahedra in condensed silicates and aluminates can be 4-connected. This can be understood as the preservation of one phosphorus–oxygen double bond within the phosphate unit in all accessible condensed structures. These connectivity patterns allow silicates and aluminates to adopt more condensed anionic networks than those in ultraphosphates, such as pure 3-connected and 4-connected anionic networks.^21,22^ Most tectosilicates and some alkali polyaluminates adopt 4-connected nets.^23−25^ There are a large number of 4-connected porous tectosilicates with large cavities or tunnels, such as clathrasils and zeolites.^21,26^ However, in general, compared with 2,3-connected nets, the pure 3-connected and 4-connected nets prefer the most stable dense networks with narrow interstices, which are unfavorable for forming 3D ion migration pathways. A paradigmatic example is γ-LiAlO_2_, which is one of the materials used as a coating for lithium-conducting electrodes and adopts a dense 4-connected tetrahedral network with small channels and void spaces (Figure 1f) that are associated with the extremely low Li-ion conductivity (below 10^–15^ S cm^–1^ at room temperature).^27−29^ In contrast, the more open 2,3-connected anionic networks of ultraphosphates are of interest because they could create large void spaces that form favorable channels or interstitial positions for cations to diffuse through the structure. For example, the ultraphosphate layer could provide large PO_4_^3–^ based rings, such as 16-membered rings in the P_4_O_11_^2–^ layer (Figure 1g), 20-membered rings in the P_5_O_14_^3–^ layer, and 14-membered rings in the P_6_O_17_^4–^ layer.^15,30,31^

This gap in our understanding of phosphate chemistry can be seen in the recent development of all-solid-state Li-ion batteries (ASLiBs), which has relied on the discovery of superionic conductors to act as solid-state electrolytes (SSEs) in bulk and thin film form.^32,33^ To date, SSEs with room temperature ionic conductivities close to or even surpassing those of organic liquid electrolytes (around 10^–3^ S cm^–1^) have been achieved in some oxide- and sulfide-based materials.^34−40^ ASLiBs can suffer from high interfacial resistance even when such highly conductive SSEs were used, which is largely due to the poor contact or incompatibility between SSEs and electrodes.^41^ Introducing a buffer layer at the cathode/SSE interface has been proposed as an effective method to suppress growth of the interfacial resistive layer. A range of cathode coatings have been investigated, and some of them have shown significant improvement of electrochemical properties of ASLiBs, including Li_4_Ti_5_O_12_,^42^ LiNbO_3_,^43^ LiTaO_3_,^44^ LiAlO_2_,^45^ Li_2_SiO_3_,^46^ Li_3_BO_3_–Li_2_CO_3_,^47^ and Li_2_ZrO_3_.^48^ However, due to their limited ionic conductivities or oxidative stability, the electrode resistance can be dominated by the coating layer itself, which hinders the further improvement of the electrochemical performance of the ASLiBs.^49,50^

Phosphates have been proposed as both SSEs and coating materials to develop ASLiBs. P is earth-abundant and phosphates have good overall characteristics, such as a wide electrochemical window, good chemical stability, acceptable ionic conductivity, and negligible electronic conductivity, which are fundamental requirements for bulk and thin film SSEs or coating materials. Different from bulk electrolytes, thin film electrolytes and coatings are thin layers, so they have less stringent requirements for ionic conductivity than the bulk electrolyte. Li ion conductors with a room temperature conductivity of around 10^–6^ S cm^–1^ can be used as thin film electrolytes. For example, lithium phosphorus oxynitride (LiPON) has been widely used in commercial solid-state thin film batteries due to its acceptable room temperature ionic conductivities ((1–3) × 10^–6^ S cm^–1^) and excellent long-term stability in contact with Li metal.^51−53^ Compared with the electrolytes, coating materials have stricter requirements in terms of chemical and electrochemical stabilities. A practical cathode coating should be chemically compatible with both the cathode, and the SSE and should have an electrochemical window that spans the cathode operating voltage and overlaps with the electrochemical window of the electrolyte. Li_3_PO_4_ is considered one of the promising coatings because of its excellent chemical and electrochemical stabilities and convenient sources without complex processing.^49,50,54−57^ A recent report on computational evaluation of potential coatings identified that phosphates exhibit higher oxidation stability limits and lower reactivity with thiophosphate electrolytes than conventional ternary metal oxide coatings such as LiTa_3_O_8_, LiNbO_3_, LiAlO_2_, and that the oxidation stability increases as the phosphate condensation increases.^58^ As a result, lithium polyphosphate LiPO_3_ has been highlighted as one of the appealing candidate for cathode coatings, as it has a higher oxidation limit than that of Li_3_PO_4_. However, both Li_3_PO_4_ and LiPO_3_ still suffer from very limited Li-ion conductivities (∼10^–16^–10^–19^ S cm^–1^ for γ-Li_3_PO_4_ and ∼10^–19^ S cm^–1^ for LiPO_3_ at room temperature).^59−61^

Lithium ultraphosphates would offer structural diversity to address this problem. However, although the structures and functional properties of alkali metal orthophosphates, polyphosphates, and cyclophosphates have been well studied in the literature,^1,2,60,62^ there are very few studies on alkali metal ultraphosphates. Apart from some Raman and nuclear magnetic resonance (NMR) spectroscopic studies on low alkali phosphate glasses that have provided evidence for the existence of ultraphosphate anions in the presence of alkali metals,^63−65^ the structures of crystalline ternary lithium ultraphosphates and their functional properties are unknown.

The unique topological arrangement of PO_4_^3–^ tetrahedra in ultraphosphates may favor the mobility of the cations, thereby offsetting low Li ion content and leading to potential increases in ionic conductivity. It is therefore important to identify lithium ultraphosphates and explore the arising electrochemical stability and ion mobility when the Li/P ratio falls below that in previously studied Li–P–O ternaries.^4,60,66,67^ We report two lithium ultraphosphates Li_3_P_5_O_14_ and Li_4_P_6_O_17_ with different structural motifs, synthesized using solid-state methods. Li_3_P_5_O_14_ shows the highest ionic conductivity and lowest activation energy among reported ternary oxides in the Li–P–O phase field, which can be attributed to its unique 3D lithium migration pathways evidenced through bond valence sum (BVS) mapping.^68^ Li_3_P_5_O_14_ is chemically stable in air. Both Li_3_P_5_O_14_ and Li_4_P_6_O_17_ have relatively high computed thermodynamic stability against oxidation: Li_3_P_5_O_14_ is predicted to be stable up to 4.8 V, significantly higher than other solid electrolytes,^58,69−72^ including other phosphates and the commercialized LiPON thin film electrolyte with comparable lithium conductivity.^58,69,70^

## 2 Experimental Section

### 2.1 Synthesis

#### 2.1.1 Materials

Li_2_O (97%) and P_2_O_5_ (≥98.0%) purchased from Sigma-Aldrich were dried overnight under vacuum (10^–4^ mbar) at room temperature before being transferred into an Ar-filled glovebox. All precursors and resulting powders were handled in an Ar-filled glovebox (O_2_ < 0.1 ppm, H_2_O < 0.1 ppm).

#### 2.1.2 Synthesis of Li_3_P_5_O_14_

The synthesis of Li_3_P_5_O_14_ was performed in two steps: First, Li_2_O and P_2_O_5_ with a molar ratio of 3:5 were mixed via ball milling under an inert argon atmosphere in a Fritsch Pulverisette 6 planetary system using a zirconia bowl (45 mL) and seven balls (15 mm in diameter) for 2 h at 300 rpm. The resulting powders were transferred to an alumina crucible, which was then sealed in a silica tube under vacuum (<10^–4^ mbar). The tube was heated to 573 K at a ramp rate of 1 K min^–1^, held at 573 K for 24 h, and cooled to room temperature at a rate of 1 K min^–1^. In a second step, the resulting powder was manually ground with an agate pestle and mortar for 30 min under an inert argon atmosphere and transferred to an alumina crucible and then sealed in an evacuated silica tube before heating to 723 K for 24 h with a heating and cooling rate of 5 K min^–1^. The resulting powder was then manually ground in a pestle and mortar and then pelleted, sealed in evacuated silica tube, and sintered at 743 K for 24 h to prepare dense pellets for further characterization. Single crystals of Li_3_P_5_O_14_ were grown by heating the above-prepared powder sample in an evacuated silica tube to 743 K for 24 h with a slow cooling rate of 1 K min^–1^.

#### 2.1.3 Synthesis of Li_4_P_6_O_17_

Single crystals of Li_4_P_6_O_17_ were obtained with two heat treatments. A mixture with a 3:5 ratio of Li_2_O and P_2_O_5_ was mixed with ball milling (same conditions as Li_3_P_5_O_14_), then sealed in an evacuated silica tube, and then heated to 773 K at a high ramp rate of 10 K min^–1^. After cooling, the resulting powder was then reground, pelleted, and sealed in an evacuated silica tube and heated at 673 K for 6 days with a ramp rate of 1 K min^–1^. Using this method, a sample with Li_3_P_5_O_14_ and Li_4_P_6_O_17_ crystals was obtained (Figure S4).

The same synthetic procedure used in Li_3_P_5_O_14_ synthesis was also used in an attempt to prepare pure bulk samples of Li_4_P_6_O_17_ with a stoichiometric ratio of Li_2_O and P_2_O_5_ (2:3). The synthesis was attempted with varying reaction temperatures (673–773 K) and reaction times (1–6 days); however, Li_3_P_5_O_14_ and LiPO_3_ always formed as the majority phases.

### 2.2 Characterization

#### 2.2.1 Single Crystal X-ray Diffraction

The single crystals suitable for structure determination of the title compounds were selected under a polarizing microscope and then mounted on a Rigaku MicroMax-007 HF X-ray generator equipped with a Mo Kα rotating-anode microfocus source and a Saturn 724+ detector. The crystals were kept at 293 K for Li_3_P_5_O_14_ and 100 K for Li_4_P_6_O_17_ during data collection. Li_3_P_5_O_14_ was also studied by single crystal XRD on beamline I19, Diamond Light Source, Didcot, U.K. using silicon double crystal monochromated synchrotron radiation (λ = 0.6889 Å, Pilatus 2 M detector).^73^ The synchrotron data were collected at 100 K. Cell refinement and data reduction were carried out with the CrysAlisPro171.40_64.42a software.^74^ The structures were solved using the Intrinsic Phasing method provided by the ShelXT^75^ structure solution program and refined with the ShelXL^76^ refinement package using Least Squares minimization, interfaced through Olex2.^77^ For Li_3_P_5_O_14_, final anisotropic atomic refinements converged to *R*_1_ = 0.0378 and *wR*_2_ = 0.0834 for reflections with *I* ≥ 2σ(*I*) for lab data, and *R*_1_ = 0.0343 and *wR*_2_ = 0.0851 for reflections with *I* ≥ 2σ(*I*) for synchrotron data. The Flack parameter is 0.06(4) for lab data and −0.03(3) for synchrotron data. For Li_4_P_6_O_17_, final anisotropic atomic refinements converged to *R*_1_ = 0.0269 and *wR*_2_ = 0.0720 for reflections with *I* ≥ 2σ(*I*). Both structure models were checked using the ADDSYM subroutine of PLATON^78^ to confirm that no additional symmetry elements could be applied to the model. The structure description and analysis are based on lab data. Crystallographic data and structural refinements are summarized in Table S1. The final refined atomic positions, isotropic thermal parameters, and anisotropic displacement parameters of each atom are illustrated in Tables S2–S5. Selected bond distances and angles are given in Tables S6–S9.

#### 2.2.2 Powder X-ray Diffraction

Routine assessment of sample purity was carried out using a Bruker D8 Discover diffractometer with monochromatic Cu radiation (Kα_1_, λ = 1.54056 Å) in Debye–Scherrer transmission geometry with sample powders sealed in 0.5 mm diameter borosilicate glass capillaries. All experiments were performed at room temperature. Pawley and Rietveld refinements were carried out using TOPAS Academic. Pawley fits were performed, refining the lattice parameters and the background using a Chebyshev function with 12 parameters. The peak shape was modeled using a pseudo–Voigt function. Rietveld refinements were performed with the single crystal model as a starting point without refinement of lattice sites.

#### 2.2.3 AC Impedance Spectroscopy

A pellet of Li_3_P_5_O_14_ with a thickness of 1.46 mm was used for AC impedance measurements with a Au|Li_3_P_5_O_14_|Au configuration. The pellet was made by uniaxially pressing 153 mg of material in an 8 mm cylindrical steel die at a pressure of 975 MPa (5 tons). The pellet was then sintered in an evacuated silica tube at 743 K for 24 h. A pellet with relative density of 85% was obtained. Gold electrodes were subsequently sputter-coated onto both sides of the pellet using a Q150R Plus - Rotary Pumped Coater. AC impedance measurements were performed using a custom-built sample holder, in the temperature range 303–573 K, using an impedance analyzer (Keysight Technologies E4990A) in the frequency range from 2 MHz to 20 Hz (with an amplitude of 100 mV). All procedures and measurements were carried out in an Ar-filled glovebox. The geometry-normalized impedance spectra were fitted with equivalent circuits using ZView2 software.^79^

#### 2.2.4 DC Polarization

A pellet that had been used for AC impedance measurement was then used for DC polarization measurements with a Au|Li_3_P_5_O_14_|Au configuration under a 1 V DC bias potential at four different temperatures (303, 373, 473, 573 K).

#### 2.2.5 Electrochemical Li Plating/Stripping

Symmetric Li|Li_3_P_5_O_14_|Li cells were assembled inside an Ar-filled glovebox (O_2_, H_2_O ≤ 0.1 ppm). Li_3_P_5_O_14_ pellets were polished to a thickness of *ca*. 0.3 mm. Two pieces of Li discs (99.9%, 0.38 mm thickness, Sigma-Aldrich) were polished and pressed onto Ni discs (99.95%, Advent RM). The Ni/Li|Li_3_P_5_O_14_|Li/Ni stack was carefully aligned, compressed, and sealed inside a two-electrode Swagelok cell. The galvanostatic plating/stripping tests were performed at 298 K at a current density of 2.5 μA cm^–2^ (30 min per half-cycle) using an MPG2 potentiostat (BioLogic Science Instruments).

#### 2.2.6 Nuclear Magnetic Resonance (NMR) Spectroscopy

Variable temperature ^7^Li NMR experiments were recorded with a 4 mm HX High Temperature MAS Probe on a 9.4 T Bruker Avance III HD spectrometer under static conditions with the X channel tuned to ^7^Li at ω_0_/2π (^7^Li) = 156 MHz. The sample was sealed in a glass ampoule, and the spectra were recorded with a pulse length of 1.5 μs at a radio frequency (rf) field amplitude of ω_1_/2π = 83 kHz and referenced to 10 M LiCl in D_2_O at 0 ppm. Temperature calibrations were performed using the chemical shift thermometers Pb(NO_3_)_2_ using ^207^Pb NMR^80^ and CuI and CuBr using ^63^Cu NMR.^81^ The errors associated with this method were calculated using the isotropic peak line broadening and range from 5 to 20 K.

#### 2.2.7 Microscopy

Scanning Electron Microscopy (SEM) and Energy Dispersive X-ray spectroscopy (EDX) were performed with a TESCAN S8000 equipped with an EDX detector from Oxford instruments. Powder samples were dispersed on a carbon tape attached to an alumina stub and coated with a thin film of carbon before performing imaging and EDX. Transmission Electron Microscopy (TEM) and Selected Area Electron Diffraction (SAED) were performed on a JEOL 2100+ using a double tilt holder. Powder samples were dispersed on a carbon coated copper TEM grid. In order to prevent the samples being exposed to air, samples were prepared inside an argon-filled glovebox in which the amount of O_2_ and moisture was kept <0.1 ppm. Samples were transferred into the TEM using a double tilt, beryllium vacuum transfer holder from Gatan and into the SEM using a modified transfer holder from Quantum. Electron diffraction patterns for Li_3_P_5_O_14_ were simulated and compared with the experimental ones using Single Crystal software from CrystalMaker Software Ltd.

#### 2.2.8 Differential Thermal Analysis (DTA)

Differential thermal analysis (DTA) and thermogravimetric analysis (TGA) were carried out on a TA Instruments Q600 Thermogravimetric Analyzer. Data were recorded on heating to 873 K and then cooling to RT with a heating rate of 10 K min^–1^ and cooling rate of 5 K min^–1^ under a constant flow rate of N_2_ (50 mL min^–1^).

#### 2.2.9 Computational Details

The thermodynamic stabilities of Li_3_P_5_O_14_ and Li_4_P_6_O_17_ were assessed by first optimizing geometries of their structures, and then comparing the calculated values of their enthalpies with respect to the convex hull built for all known compositions in the Li–P–O phase field. All calculations were performed with DFT as implemented in VASP-5.4.4^82^ with the plane-waves approach, PBE exchange-correlation functional, and projected augmented wave method for treating core electrons.^83^ During the geometry optimization, interatomic forces were decreased to less than 10^–3^eV Å^–1^, and changes of total energy in self-consistent runs were converged to less than 10^–10^ eV. The plane waves were sampled at 5 × 5 × 5 k-points mesh with energy cut off at 700 eV.

The electrochemical stability window was calculated with an approach analogous to the Li-grand canonical phase diagram.^70,84^ For each electrolyte compound (EC), we construct decomposition reactions
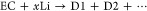
1for all possible values *x*_*i*_ and D1, D2, . . .decomposition products, and for each such reaction *i* we compute the corresponding equilibrium potential Φ_*eq*,*i*_ by the Nernst equation:^85^
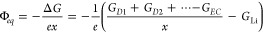
2where Gibbs energies of the compounds, *G*_D__1_, *G*_D__2_, etc., are approximated as their enthalpies, computed with DFT as described above. The electrochemical window is then approximated by the phase stability window (not taking into account electron and ion transfer), with the potential limits calculated as max({ Φ_*eq*,*i*_ | *x*_*i*_ > 0}) for reduction and min({ Φ_*eq*,*i*_ | *x*_*i*_ < 0}) for oxidation.

## 3 Results and Discussion

### 3.1 Structure Description

#### 3.1.1 Structure of Li_3_P_5_O_14_

**Figure 2:**
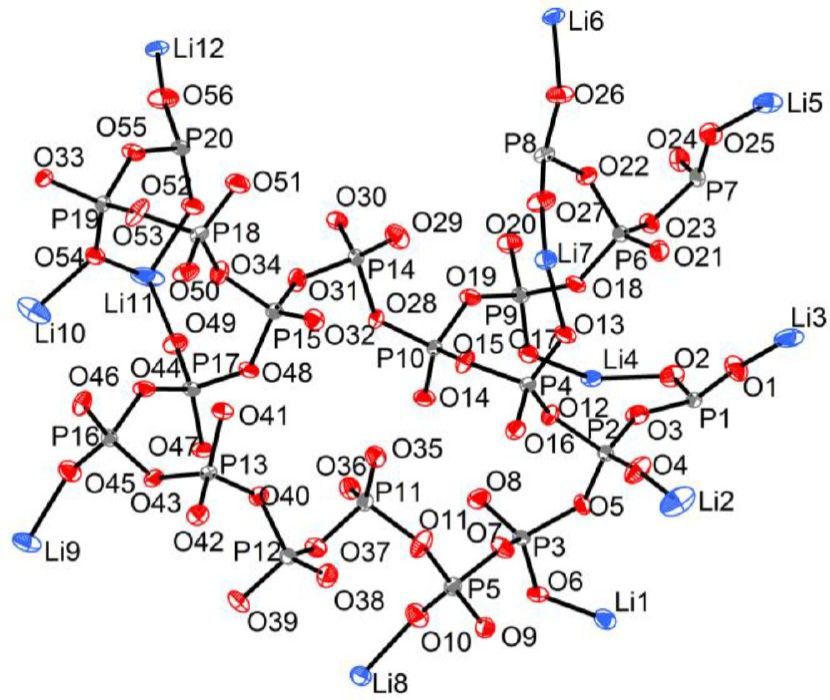
Asymmetric unit of Li_3_P_5_O_14_ with displacement ellipsoids shown at 50% probability (293 K). All the atoms occupy 4a sites and are ordered. Li, P and O atoms are shown in blue, gray, and red, respectively.

Li_3_P_5_O_14_ crystallizes in the monoclinic space group *Cc* (no. 9) with lattice parameters *a* = 33.3764(13) Å, *b* = 11.0005(2) Å, *c* = 15.0881(6) Å, β = 128.046(6)°, and *V* = 4362.6(4) Å^3^. There are 16 formula units per unit cell. In the asymmetric unit, there are 12 crystallographically distinct Li atoms, 20 P atoms, and 56 O atoms (Figure 2). The 12 Li atoms occupy 11 distorted tetrahedral sites and one distorted square pyramid site with Li–O bond lengths in the range 1.807(10)–2.436(13) Å and O–Li–O bond angles in the range 80.0(4)°–150.7(5)°. All P atoms are in fourfold coordination environments with O atoms forming distorted PO_4_^3–^ tetrahedra with P–O bond lengths and O–P–O bond angles of 1.433(4)–1.631(3) Å and 97.19(18)°–124.2(2)°, respectively. These values agree with those commonly observed in other phosphates, particularly condensed phosphates.^86−90^ Li_3_P_5_O_14_ has 88 unique atoms in the asymmetric unit, which is the largest number for all reported crystallographically characterized lithium phosphates. The previous most crystallographically complex lithium phosphate, from the 11 ternary lithium phosphates (Table S10), is the monoclinic polyphosphate LiPO_3_ (Pc) with 50 atoms in the asymmetric unit. Compared with unbranched 0D rings and finite ribbon or 1D infinite ribbons in these known ternary lithium phosphates, the branching PO_4_^3–^ tetrahedra in Li_3_P_5_O_14_ condense the PO_4_^3–^ tetrahedra in 2D to form an ultraphosphate layer.

**Figure 3:**
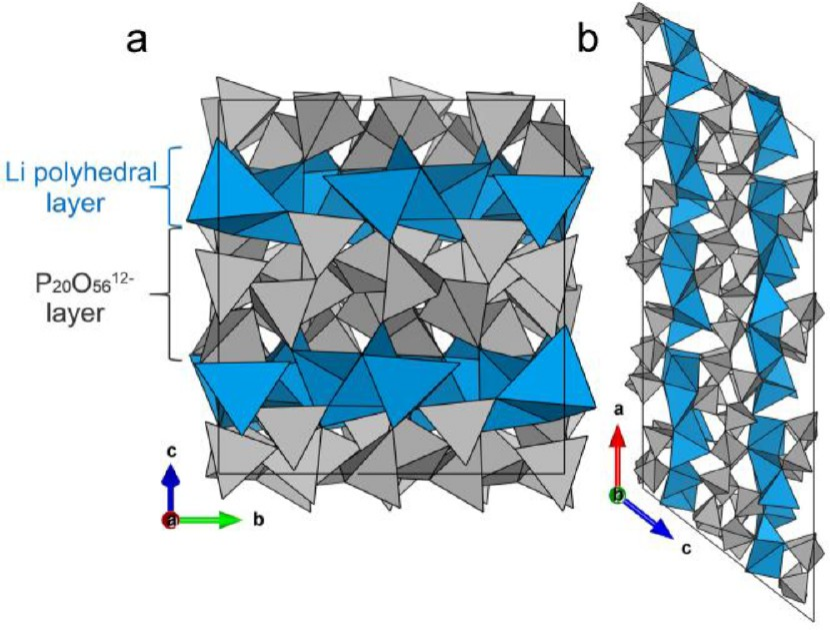
Unit cell of Li_3_P_5_O_14_ with projections along the (a) *a* and (b) *b* axes, respectively, showing the alternating stacking arrangement of the ultraphosphate layers and the Li polyhedral layers along *c* axis. PO_4_^3–^ tetrahedra shown in gray, and Li polyhedra in blue.

Li_3_P_5_O_14_ is a layered structure built from infinite ultraphosphate P_20_O_56_^12–^ layers with 12-membered corrugated P_12_O_36_^12–^ rings constructed from corner-sharing PO_4_^3–^ tetrahedra, alternately stacked with Li polyhedral layers along the *c* axis (Figures 3a and 3b). The charge compensating Li cations are located between P_20_O_56_^12–^ layers, forming bonds to four or five oxide ions in these adjacent layers. There is no P–O–P linkage between layers.

**Figure 4:**
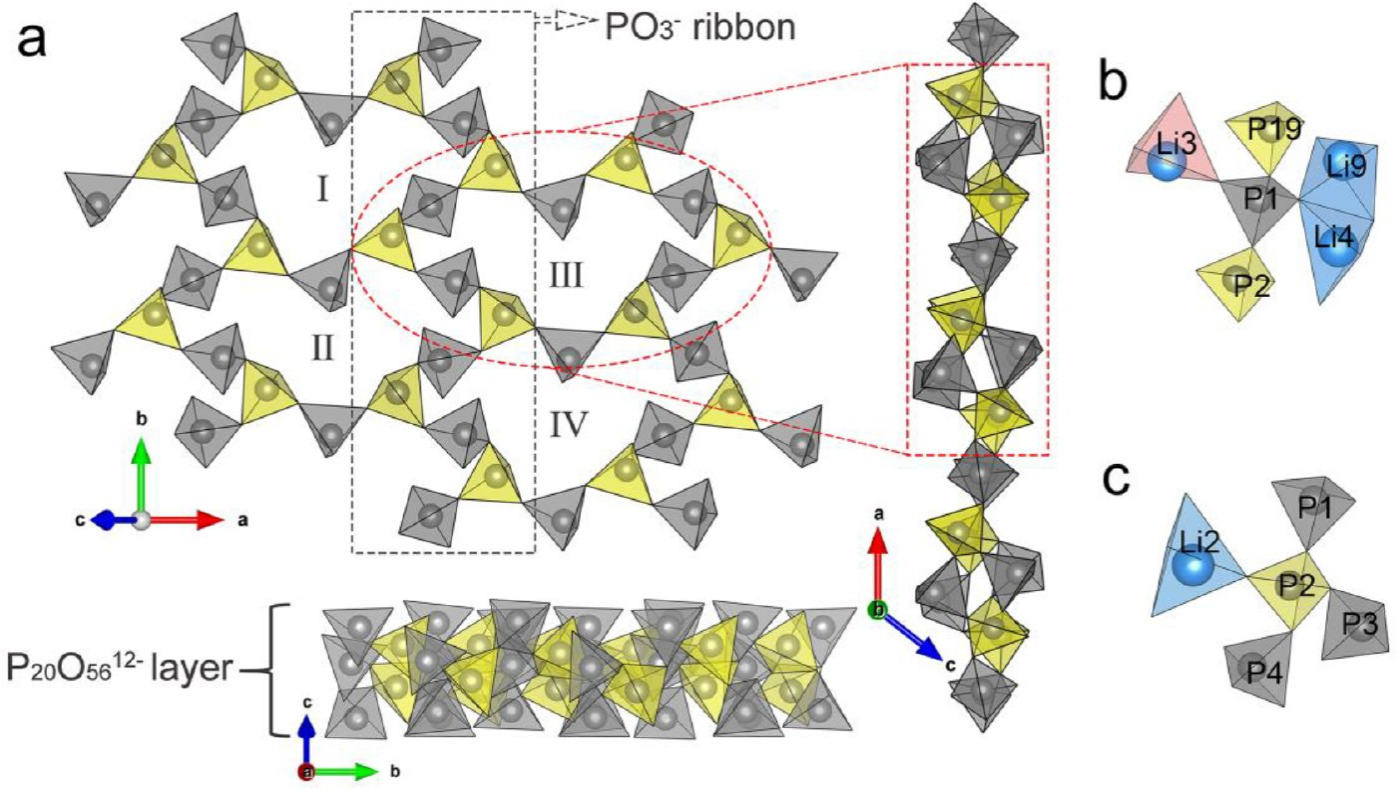
(a) Infinite ultraphosphate layer of P_20_O_56_^12–^ in Li_3_P_5_O_14_ with four crystallographically different 12-membered corrugated rings of alternating internal and branching PO_4_^3–^ tetrahedra. Three projections are shown: onto *ab* plane and perpendicular thereto (along *a* and *b* axes). Internal PO_4_^3–^ tetrahedra are colored in gray, and branching PO_4_^3–^ tetrahedra are colored in yellow. The coordination environments of (b) internal P1 tetrahedra and (c) branching P2 tetrahedra are shown. The Li polyhedra from the type A Li_6_O_16_^26–^ chain are colored in red, and those from the type B Li_6_O_16_^26–^ chain are colored in blue.

The P_20_O_56_^12–^ layers adopt a 2,3-connected anionic network constructed from alternating internal and branching PO_4_^3–^ tetrahedra (Figure 4a) in a 3:2 ratio. As observed for reported condensed phosphates, the bonds from P atoms to the bridging O atoms are significantly longer compared with those to the terminal O atoms in Li_3_P_5_O_14_. The bond lengths of terminal and bridging P–O are in the range 1.433(4)–1.482(3) and 1.542(4)–1.631(3) Å, respectively. The P_20_O_56_^12–^ layers consist of a succession of 12-membered corrugated P_12_O_36_^12–^ rings of PO_4_^3–^ tetrahedra, which provide pathways for ion transport between adjacent Li polyhedral layers. There are four crystallographically distinct P_12_O_36_^12–^ rings, which are similar in size and shape, and each is connected to six adjacent rings through branching PO_4_^3–^ tetrahedra to form the infinite ultraphosphate layer. Alternatively, this strongly anionic layer can also be described as built up by a set of zigzag infinite PO_3_^–^ ribbons parallel to the *b* axis constructed from alternating internal and branching PO_4_^3–^ tetrahedra, and then interconnected by internal PO_4_^3–^ tetrahedra, as shown in Figure 4a. The P_20_O_56_^12–^ layer is analogous to some phyllosilicate anions with 2,3-connected nets, such as single layer Si_3_O_8_^4–^ in Na_2_ZnSi_3_O_8_.^20^ The one major difference is the proportion of branching tetrahedra in their layer, which is 2/3 in Na_2_ZnSi_3_O_8_, higher than 2/5 of Li_3_P_5_O_14_, indicating the Na_2_ZnSi_3_O_8_ anions have higher degrees of condensation than that of Li_3_P_5_O_14_.

The O in branching PO_4_^3–^ tetrahedra have different connectivity with Li than those in the internal tetrahedra. Internal PO_4_^3–^ tetrahedra share two O atoms with two of the neighboring branching PO_4_^3–^ tetrahedra, the remaining two O atoms are involved in coordination to three to four different Li polyhedra that are either from two adjacent Li polyhedral layers or from two different types of Li_6_O_16_^26–^ chains in the same Li polyhedral layer (Figure 4b). In contrast, branching PO_4_^3–^ tetrahedra already share three of four O atoms with neighboring internal PO_4_^3–^ tetrahedra, so only one O atom is available to coordinate with one or two Li polyhedra (Figure 4c).

**Figure 5:**
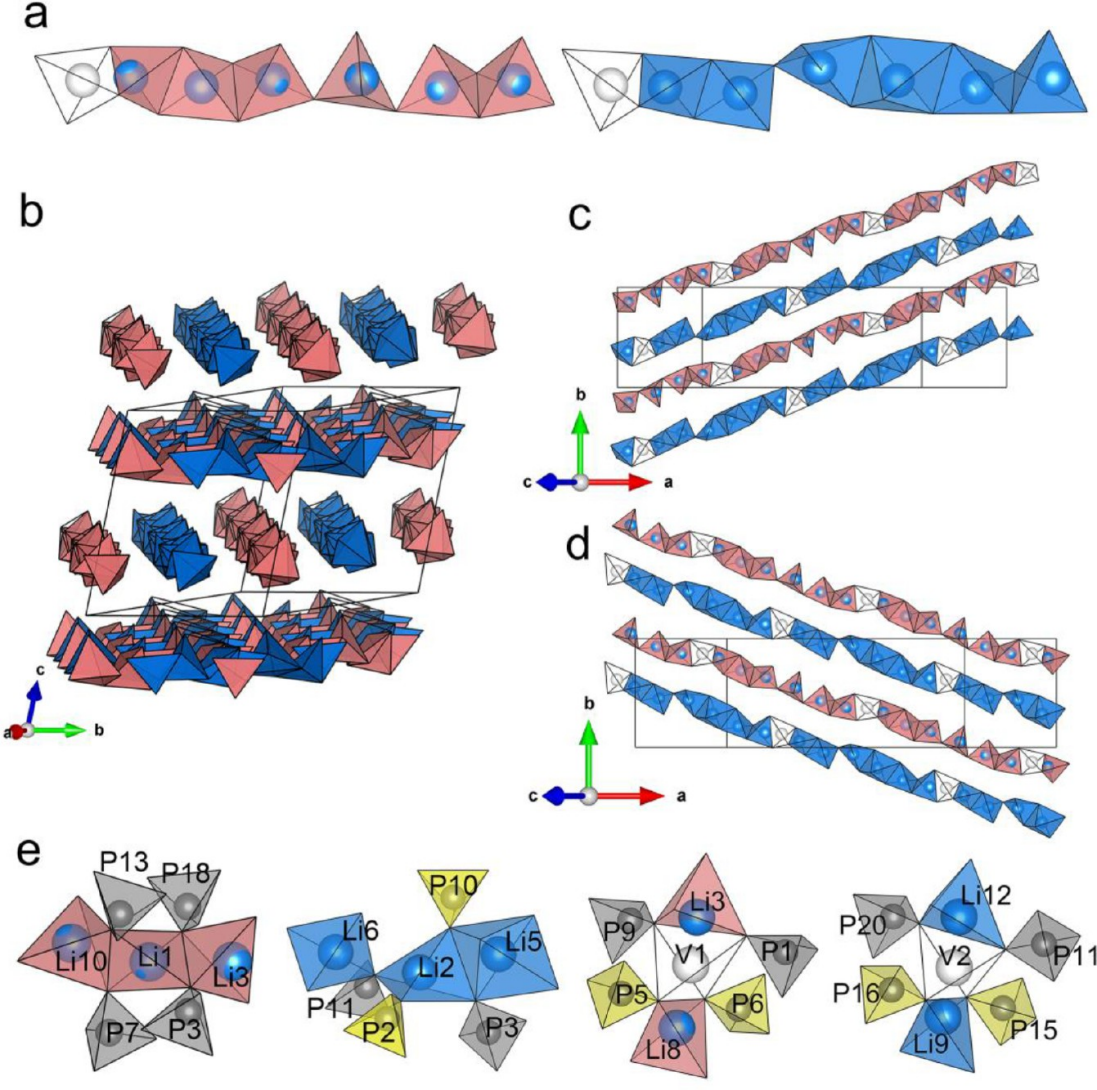
Arrangement of lithium in Li_3_P_5_O_14_. (a) The two types of Li_6_O_16_^26–^ chains, type A (red) and type B (blue), with different connection modes along with two distinct vacant tetrahedral sites at the end of them. (b) Arrangement of Li polyhedra viewed approximately along [100], showing the alternating arrangement of Li polyhedral chains. (c) Li_6_O_16_^26–^ chains along [110] in layer a and (d) along [110] in layer b. (e) Coordination environments of two Li occupied sites, Li1 and Li2, and two distinct vacant tetrahedral sites, V1 and V2. White tetrahedra show the vacant sites; vacant site 1 (V1) is at the end of the type A Li_6_O_16_^26–^ chain, and V2 is at the end of the type B Li_6_O_16_^26–^ chain. Gray and yellow tetrahedra show the internal and branching PO_4_^3–^ tetrahedra respectively; red and blue tetrahedra show the LiO_4_^7–^ tetrahedra in type A and B Li_6_O_16_^26–^ chains, respectively. P, gray; Li, blue.

These strong covalently bonded P_20_O_56_^12–^ layers produce a unique topology for the Li sublattices: two types of finite Li polyhedral Li_6_O_16_^26–^ chains with comparatively short Li–Li distances (2.581–3.235 Å in the Li_6_O_16_^26–^ chains) terminated with two distinct vacant tetrahedral sites, V1 and V2 (Figure 5a). These ordered Li_6_O_16_^26–^ chains are isolated from each other. The type A Li_6_O_16_^26–^ chain is made up of six crystallographically distinct corner- and edge-shared distorted tetrahedra. The type B Li_6_O_16_^26–^ chain consists of five distorted tetrahedra and an Li5 distorted square pyramid connected by corner and edge sharing. These two types of Li_6_O_16_^26–^ chains are alternately arranged parallel to the *ab* plane forming Li polyhedral layers, as shown in Figure 5b. There are two distinct types of Li polyhedral layer in Li_3_P_5_O_14_ that are alternately stacked along the *c* axis. The Li_6_O_16_^26–^ chains in the layer a lie along [110] (Figure 5c) while they are along [110] in layer b (Figure 5d). These Li polyhedral layers are further alternately stacked with infinite ultraphosphate layers along *c* to form a 3D framework. Similar to the Li-occupied sites, the two vacant tetrahedra, V1 and V2, are coordinated by four PO_4_^3–^ tetrahedra by corner sharing (two internal PO_4_^3–^ tetrahedra and two branching PO_4_^3–^ tetrahedra), and two LiO_4_^7–^ tetrahedra by edge sharing, as shown in Figure 5e.

#### 3.1.2 Structure of Li_4_P_6_O_17_

**Figure 6:**
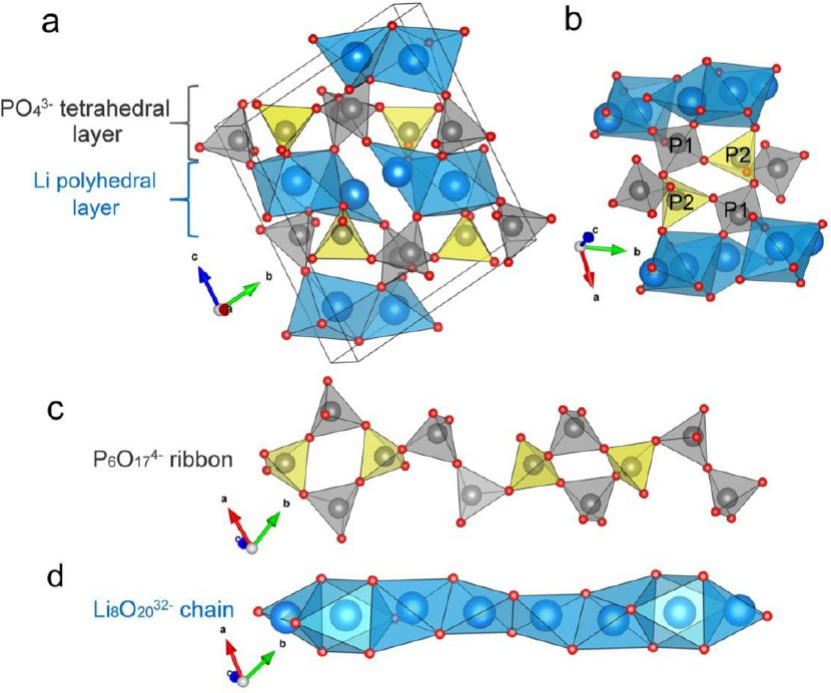
Crystal structure of Li_4_P_6_O_17_. (a) Unit cell of Li_4_P_6_O_17_ based on alternating stacked PO_4_^3–^ tetrahedral and Li polyhedral layers along the [012] direction. (b) Internal (P1) and branching (P2) tetrahedral environments. (c) Infinite loop-branched P_6_O_17_^4–^ single ribbon with 4-membered P_4_O_12_^4–^ rings. These P_6_O_17_^4–^ ribbons are arranged parallel to each other to form the PO_4_^3–^ tetrahedral layers. (d) The finite Li_8_O_20_^32–^ chain consisting of edge- and face-sharing of Li polyhedra. The Li_8_O_20_^32–^ chains are parallel to the P_6_O_17_^4–^ ribbons and arranged in layers, which are stacked alternately with PO_4_^3–^-based layers along the [012] direction. Li, blue; P, gray; O, red. Gray tetrahedra and yellow tetrahedra mark the internal and branching PO_4_^3–^ tetrahedra, respectively. Li polyhedra are colored in blue.

Li_4_P_6_O_17_ crystallizes in the triclinic space group *P*1 (no. 2) with two formula units per unit cell. The unit cell parameters are *a* = 7.3721(3) Å, *b* = 8.9291(3) Å, *c* = 10.8581(4) Å, α = 79.596(3)°, β = 81.961(3)°, γ = 70.413(3)°, *V* = 659.89(4) Å^3^. In the asymmetric unit, there are four crystallographically distinct Li atoms, six P atoms, and 17 O atoms (Figure S1). All the atoms occupy 2i sites and are ordered. In the structure of Li_4_P_6_O_17_, six distinct PO_4_^3–^ tetrahedra share vertices to form a loop-branched P_6_O_17_^4–^ single ribbon with the periodic repetition of 12 PO_4_^3–^ tetrahedra. These P_6_O_17_^4–^ ribbons are arranged in layers parallel to the (012) plane, alternating stacked with Li polyhedral layers along the [012] direction, as shown in Figure 6a. There are two distinct P_4_O_12_^4–^ 4-membered rings, which are arranged alternately in P_6_O_17_^4–^ ribbons, as shown in Figure 6c. The internal symmetry of P_4_O_12_^4–^ rings is 1 as observed in some reported cyclophosphates.^3^ The P_4_O_12_^4–^ 4-membered rings in Li_4_P_6_O_17_ are interconnected by P_2_O_7_^4–^ groups in 1D through branching PO_4_^3–^ tetrahedra, unlike the isolated rings in cyclophosphates.

The ratio of internal to branching PO_4_^3–^ tetrahedra is 4:2 in Li_4_P_6_O_17_, which is higher than 3:2 in Li_3_P_5_O_14_, indicating the anions in Li_3_P_5_O_14_ are more condensed than those of Li_4_P_6_O_17_. The terminal and bridging P–O bond lengths are 1.4623(13)–1.4575(13) Å, and 1.5576(13)–1.5782(13) Å in branching tetrahedra, and 1.4662(13)–1.4686(13) and 1.5897(13)–1.6358(13) in internal tetrahedra, which are comparable to those in Li_3_P_5_O_14_ (Tables S6 and S7).

**Figure 7:**
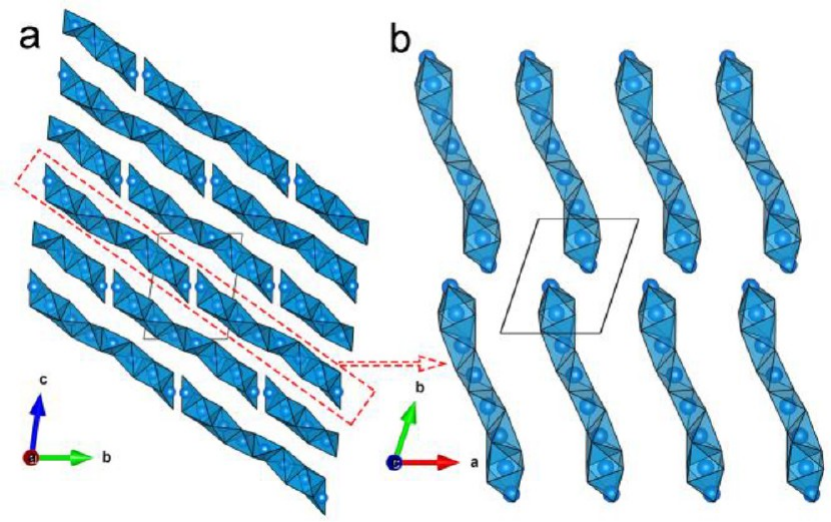
(a) Alternating arrangement of Li_8_O_20_^32–^ chains viewed along [100]. (b) The Li polyhedral layer consisting of a series of Li_8_O_20_^32–^ chains arranged in parallel to each other.

Similar to Li_3_P_5_O_14_, the O in branching PO_4_^3–^ tetrahedra have different involvement in coordination to Li from those in internal PO_4_^3–^ tetrahedra. Internal PO_4_^3–^ tetrahedra share two of their O atoms with two of the neighboring PO_4_^3–^ tetrahedra and two in coordination to three to four Li polyhedra. Branching PO_4_^3–^ tetrahedra share three of their O atoms with neighboring internal PO_4_^3–^ tetrahedra and one with two neighboring Li polyhedra, as shown in Figure 6b. The Li polyhedral layers are constructed from a set of finite Li_8_O_20_^32–^ chains arranged in parallel to each other and stacked along the [012] direction, as shown in Figures 6d and 7. The chains consist of distorted tetrahedra, octahedra, and square pyramids connected by face- and edge-sharing and are parallel to the P_6_O_17_^4–^ ribbons and are interconnected by internal PO_4_^3–^ tetrahedra.

#### 3.1.3 Structure Comparison with Other Ultraphosphates

The general anionic formula for all observed ultraphosphates is P_(*n*+2)_O_(3*n*+5)_^*n*–^, where *n* = 2, 3, 4, 5, and 6.^91,92^ With increasing *n*, the ratio of internal to branching PO_4_^3–^ tetrahedra and the average value of the negative charges per P increases, the degree of condensation of PO_4_^3–^ tetrahedra decreases, and ultraphosphates accordingly tend to adopt low-dimensional configurations. For example, the P_4_O_11_^2–^ (*n* = 2) anion adopts 2D layers, the P_5_O_14_^3–^ anion (*n* = 3) adopts both infinite 1D ribbons^93,94^ and 2D layers while only 1D ribbons are observed for P_7_O_20_^5–^ (*n* = 5)^92^ and 0D finite groups for P_8_O_23_^6–^ (*n* = 6).^6,16^ In order to meet the specific ratio of internal and branching tetrahedra, the P_5_O_14_^3–^ anion adopts an alternating arrangement of internal and branching tetrahedra, while ultraphosphates with smaller and larger *n* values feature the branching–branching and internal–internal tetrahedral connections, respectively.

**Figure 8:**
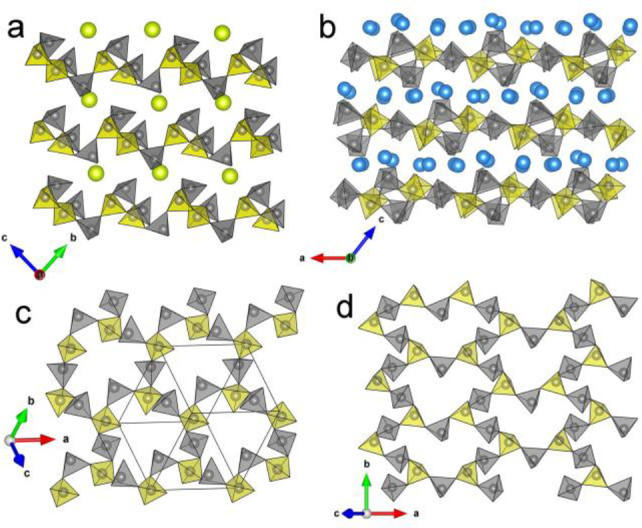
Crystal structure comparison between triclinic CeP_5_O_14_ and Li_3_P_5_O_14_. (a) Projection of the atomic arrangement in triclinic CeP_5_O_14_ along the *a* axis, showing folded P_10_O_28_^6–^ ultraphosphate layers alternately stacked with layers of cerium atoms. (b) Projection of the atomic arrangement in Li_3_P_5_O_14_ along the *b* axis, showing flatter P_20_O_56_^12–^ ultraphosphate layers alternately stacked with Li polyhedral layers. The internal organization of (c) the infinite P_10_O_28_^6–^ layers in triclinic CeP_5_O_14_ and (d) the infinite P_20_O_56_^12–^ layers in Li_3_P_5_O_14_. Compared with trivalent cerium, monovalent lithium increases the ratio of the metal cation to P, thereby reducing the folding of its ultraphosphate layers. The gray and yellow tetrahedra represent internal and branching PO_4_^3–^ tetrahedra. Phosphorus is shown as gray spheres, and cerium and lithium are shown as green and blue spheres, respectively.

The P_5_O_14_^3–^ anion could adopt a different 2D layered arrangement with different sizes of rings based on alternating arrangement of internal and branching tetrahedra. The P_5_O_14_^3–^ layers in monoclinic YP_5_O_14_ present a combination of 20- and 8-membered rings,^15^ while the P_10_O_28_^6–^ layer in triclinic CeP_5_O_14_ presents 12-membered corrugated rings, which have the same connectivity as the infinite P_20_O_56_^12–^ layers in Li_3_P_5_O_14_, as shown in Figure 8.^94^ These rings are interconnected with each other in 2D through their branching PO_4_^3–^ tetrahedra. Compared with the folded ultraphosphate layer in triclinic CeP_5_O_14_ (Figures 8a and 8c), the layers in Li_3_P_5_O_14_ are almost flat (Figures 8b and 8d), associated with the decreased ratio of metal cation to P.^23^ The mean angle of P–O–P is 135.87° for CeP_5_O_14_, slightly smaller than 136.23(26)° in Li_3_P_5_O_14_, consistent with the folding of their ultraphosphate layers.

There are only a few reported ultraphosphates with the P_6_O_17_^4–^ anion, including Ca_2_P_6_O_17_, Sr_2_P_6_O_17_, Cd_2_P_6_O_17_, and (UO_2_)_2_P_6_O_17_.^87,95,96^ The P_6_O_17_^4–^ anions in Ca_2_P_6_O_17_, Sr_2_P_6_O_17_, and Cd_2_P_6_O_17_ are characterized by infinite ultraphosphate layers constructed from 14-membered rings interconnected in 2D through their branching PO_4_^3–^ tetrahedra. The P_6_O_17_^4–^ anion in (UO_2_)_2_P_6_O_17_ has a 3D network. In contrast, Li_4_P_6_O_17_ presents an infinite 1D loop-branched P_6_O_17_^4–^ ribbon, which is the first example of a single ultraphosphate ribbon, contrasting with the double ultraphosphate ribbons in monoclinic LaP_5_O_14_ (Figure S2a) and P_7_O_20_^5–^ in CaYP_7_O_20_ (Figure S2b).

### 3.2 Synthesis and Phase Purity

To obtain phase pure Li_3_P_5_O_14_, the heating rate was controlled carefully to avoid the sublimation of P_2_O_5_ in the case of rapid heating and the presence of LiPO_3_ impurities if the heating rate was too slow. Accordingly, a two-step synthesis strategy was adopted to obtain phase pure Li_3_P_5_O_14_. For the first firing, a slow heating rate is required to avoid melting and glass transition of the mixture. For the second firing, a higher heating rate is required to reduce the content of the impurity of LiPO_3_. Figure S3 shows the powder XRD patterns for simulated and experimental data. There is a small amount of impurity of LiPO_3_ in the powder after performing the second firing at 723 K, but it was possible to obtain phase pure powder of Li_3_P_5_O_14_ by pelleting and sintering at 743 K for 24 h. Therefore, all dense pellets for further characterization were sintered at 743 K for 24 h.

**Figure 9:**
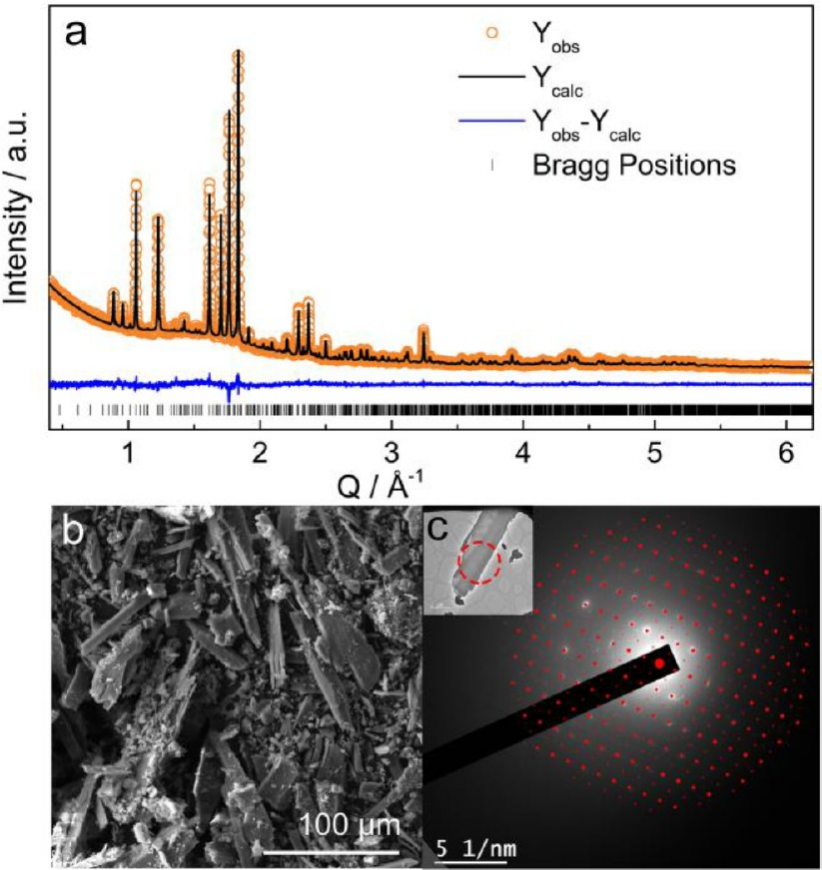
(a) Rietveld refinement against XRD pattern of as-synthesized Li_3_P_5_O_14_ with *Y*_obs_ (Orange dots), *Y*_calc_ (black line), *Y*_obs_–*Y*_calc_ (blue line), and Bragg reflections (black tick marks). *R*_wp_ = 4.72%, *R*_exp_ = 3.58%, *R*_p_ = 3.54%, χ^2^ = 1.74. (b) SEM image of as-synthesized Li_3_P_5_O_14_. (c) SAED patterns. Red dots indicate simulated pattern from single crystal structure along the [123] zone axis. Inset shows the TEM image of the particle chosen for the study; the selected area is highlighted with a red circle and carefully selected to avoid the stacking area of exfoliated layers that will lead to the presence of extra diffraction spots.

Synthesis at the composition Li_4_P_6_O_17_ was also attempted with the procedure described above to prepare pure bulk powder of Li_4_P_6_O_17_ with varying reaction temperatures (673–773 K) and reaction times (1, 2, 6 days); however, Li_3_P_5_O_14_ and LiPO_3_ always formed as the majority phases in powder synthesis. Single crystals of Li_4_P_6_O_17_ have been successfully prepared indicating that Li_4_P_6_O_17_ is stable experimentally. Failure to synthesize pure bulk powder samples of Li_4_P_6_O_17_ may reflect the kinetics of synthesis and the higher content of volatile lithium in this phase. As such, all further characterizations therefore only concern Li_3_P_5_O_14_. The PXRD data of the pure powder of Li_3_P_5_O_14_ were used to run Pawley and Rietveld refinements with the single crystal model as a starting point, as shown in Figures 9a and S5. Figure 9a shows the Rietveld refinements against laboratory powder diffraction data of the Li_3_P_5_O_14_, indicating that the obtained powder is single phase and lattice parameters agree with those of the single crystal (Tables S1 and S11). The thermal stability of Li_3_P_5_O_14_ was confirmed by TGA–DTA, which revealed no significant change in mass and no detectable phase transition in the heating processes over the temperature range 300–873 K with a melting point of 799.9(1) K (Figure S6a). Li_3_P_5_O_14_ was chemically stable when exposed to air for 1 week (Figure S6b).

SEM images of the as-synthesized Li_3_P_5_O_14_ (Figures 9b and S7) revealed particles with flattened rod-like morphologies and with a trace amount of amorphous material. The presence of elements other than Li, P, and O was ruled out based on energy dispersive X-ray (EDX) spectroscopy (Figure S8). The SAED pattern measured along the [123] direction (Figure 9c) and the opposite facet [123] (Figure S9) zone axes show good agreement with the single crystal structure of Li_3_P_5_O_14_, confirming that the particles studied correspond to the single crystal structure.

### 3.3 Theoretical Phase Stability

**Figure 10:**
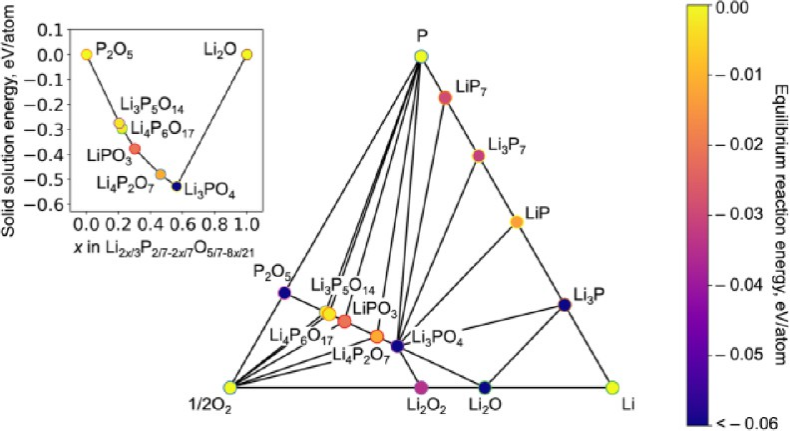
Compositions determined to be stable from calculation in the Li–P–O phase field. The structures of both Li_3_P_5_O_14_ and Li_4_P_6_O_17_ are calculated from first-principles to be stable, with negative values of formation enthalpies from the neighboring equilibrium stable compositions (as depicted by the colors of the markers). The inset illustrates the formation energy of the compositions as a solid solution between P_2_O_5_ and Li_2_O.

Theoretical thermodynamical stabilities of the synthesized phases Li_3_P_5_O_14_ and Li_4_P_6_O_17_ can be assessed from first-principles by comparison of the total enthalpies of these phases with other compounds reported in the Li–P–O phase field. There are several approaches to approximate exchange-correlation potential in density-functional theory, including the broadly applicable generalized gradient approximation (GGA) in the PBE formulation and strongly correlated and appropriated norm (SCAN) meta-GGA.^97,98^ The elemental phase diagram calculated with PBE is in a good agreement with experiment: both structures are thermodynamically stable. In the PBE approximation, the equilibrium reaction energies to form Li_3_P_5_O_14_ and Li_4_P_6_O_17_ phases from the nearest previously reported compounds in this phase diagram are negative; hence, Li_3_P_5_O_14_ and Li_4_P_6_O_17_ lower the energy level of thermodynamic stability, redefining the convex hull of the Li–P–O phase field (Figure 10). Additionally, from the compound phase diagram calculated with PBE (Figure 10, inset), the stability of these structures is also evident in terms of negative solid solution energy—when phases are represented as combinations of Li_2_O and P_2_O_5_. The compound phase diagram calculated with meta-GGA SCAN (Figure S10) underestimates the enthalpy values for Li_3_P_5_O_14_ and Li_4_P_6_O_17_ rendering them as thermodynamically unstable in contrast to experimental observations. Hence, for electrochemical stability calculations in the remainder of this work, we rely on PBE as the approach in better agreement with experiment in this case.

### 3.4 Ionic Conductivity and Electrochemical Performance

**Figure 11:**
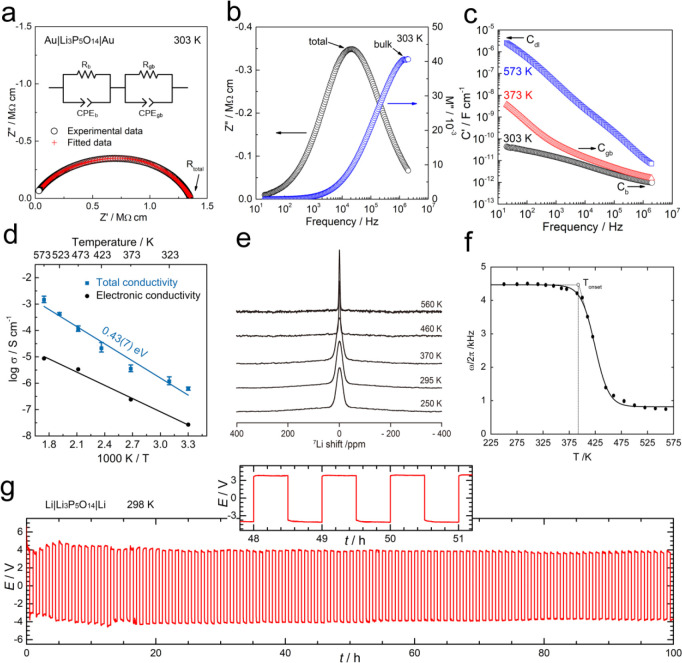
Typical impedance data, NMR, and stability of Li_3_P_5_O_14_ with lithium metal. (a) Nyquist plot obtained from a Au|Li_3_P_5_O_14_|Au cell at 303 K, and the fitting results (red cross) using the inset equivalent circuit. *R* – Resistance, *CPE* – Constant Phase Element. Impedance is normalized for geometry. (b) M″/Z′′ spectroscopic plots at 303 K. (c) Spectroscopic plots of capacitance at three different temperatures. (d) Arrhenius plots of the total conductivity measured by AC impedance spectroscopy (blue circles) and electronic conductivity measured by DC polarization (black circles). (e) ^7^Li NMR spectra as a function of temperature for Li_3_P_5_O_14_. (f) Temperature dependence of static ^7^Li NMR line width as a function of temperature. The solid line is a sigmoidal regression fit and is a guide to the eye. The onset temperature of motional narrowing (*T*_onset_) is indicated. (g) Galvanostatic plating and stripping experiment in symmetric Li|Li_3_P_5_O_14_|Li cell obtained at 298 K under a current density of ±2.5 μA·cm^–2^ (inset shows data between 48 and 51 h).

The bulk and total conductivity of Li_3_P_5_O_14_ were determined by AC impedance spectroscopy on a Au|Li_3_P_5_O_14_|Au configuration.^99^ A typical impedance data set at room temperature is shown in Figure 11a–11c. The Nyquist plot, Figure 11a, shows a broad arc, of which the low-frequency intercept on the Z′ axis gives the total resistance. On replotting the same data in the M″/Z′′ spectroscopic plot, Figure 11b, it is clear that both M″ and Z′′ have a single peak that occurs at different frequencies. The high-frequency peak in the M′′ spectrum dominates the bulk resistance, while the broad low-frequency peak in the Z′′ spectrum dominates the total resistance of the sample. Figure 11c shows capacitance data at different temperatures. At room temperature, it clearly demonstrates a high-frequency plateau with an approximate value of 10^–12^ F cm^–1^, corresponding to a permittivity of ∼10 and thus represents the bulk response. A second, poorly resolved plateau at lower frequency with a capacitance of 10^–10^ F cm^–1^ is seen at higher temperatures and is attributed to a conventional grain boundary.^99^ At the highest temperature, 573 K, C′ rises to values around 10^–5^ F cm^–1^, characteristic of a blocking double-layer capacitance at the sample–electrode interface. Although impedance measurements were not carried out at very low frequencies (e.g., 0.01 Hz) and therefore no spike was observed in the room temperature impedance plot, the low-frequency plateau at 10^–5^ F cm^–1^ is a clear evidence of blocking Li ions and indicates that the sample has ionic conductivity, which was shown more clearly by the higher temperature Nyquist plot at 373 K with the presence of a low-frequency inclined spike, as shown in Figure S11.

To extract the bulk resistance values, it was assumed initially that the high-frequency arc could be represented ideally by a series combination of two parallel *R*-*CPE* elements, as shown in Figures 11a and S11. In the equivalent circuit, the *R*_b_ and *R*_gb_ are resistors used to simulate the bulk and grain boundary resistance. The *CPE*_b_ and *CPE*_gb_ are constant phase elements used to simulate the capacitive behaviors of *R*_b_ and *R*_gb_. Final fitted parameters at 303 K are given in Table S12. The bulk and total conductivities at 303 K were estimated to be 8.5(5) × 10^–7^ and 6.2(4) × 10^–7^ S cm^–1^, respectively. These data vary with temperature and are shown in Arrhenius conductivity format in Figures 11d and S12. Linear behaviors are observed in the temperature range 303–573 K for both bulk and total conductivities with activation energies of 0.42(8) and 0.43(7) eV, respectively. In order to characterize the electronic contribution to the observed conduction, DC polarization measurements were carried out between room temperature and 573 K on a Au|Li_3_P_5_O_14_|Au configuration. Figure S13 shows the current vs time plots, and Figure 11d shows the temperature dependence of the electronic conductivity, indicating predominant ionic conduction in Li_3_P_5_O_14_. The electronic conductivity contributes less than 3% to the bulk conductivity at 303 K and less than 0.5% at 573 K.

The bulk ionic conductivity of Li_3_P_5_O_14_ at 303 K is comparable to sputtered LiPON glass thin film electrolytes ((1–3) × 10^–6^ S cm^–1^ at 298 K),^51,52,100,101^ which are routinely used in the production of thin film batteries, and the bulk-type electrolyte Li_3.6_PO_3.4_N_0.6_ (3.0 × 10^–7^ S cm^–1^ at 298 K),^102^ a crystalline polymorph of LiPON SSE with the highest ionic conductivity among all reported crystalline LiPON SSEs. The room temperature ionic conductivity of Li_3_P_5_O_14_ is higher than that of conventional crystalline metal oxide coatings, such as Li_4_Ti_5_O_12_ (5.8 × 10^–8^ S cm^–1^),^103^ LiTaO_3_ (2.1 × 10^–10^ S cm^–1^),^104^ LiNbO_3_ (∼10^–26^ S cm^–1^),^105^ and LiAlO_2_ (below 10^–15^ S cm^–1^),^27−29^ and is comparable to some of their amorphous/glass state, such as LiNbO_3_ glass (10^–5^–10^–6^ S cm^–1^).^106^ Compared to LiPON and these metal oxide coatings, Li_3_P_5_O_14_ exhibited significantly improved electrochemical oxidation stability as discussed below.

Insight into the local ionic mobility of Li_3_P_5_O_14_ was obtained through solid-state NMR. The temperature dependence of the static ^7^Li NMR spectra over the 250–560 K temperature range is shown in Figure 11e and 11f. At temperatures below ∼385 K, ^7^Li ion mobility is in the rigid lattice regime; therefore, the 1/2 ↔ −1/2 central transition is broadened by the strong ^7^Li–^7^Li homonuclear dipolar coupling and an NMR line width of ∼4.5 kHz is observed. As the temperature is increased above 385 K, where the onset of motional narrowing occurs, the line width decreases due to the increasing motion of the ^7^Li spins continuously averaging dipolar interactions. Using an expression introduced by Waugh and Fedin,^107^ relating the onset temperature of motional narrowing (*T*_onset_) with the activation energy of the diffusion process, given by eq 3:
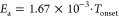
3an approximate activation energy of 0.6 eV can be estimated for Li_3_P_5_O_14_. We note that this value is only an estimate (hence no error is quoted) given the validity of the Waugh and Fedin expression, which does not take into account structural complexity^108,109^ such as that of Li_3_P_5_O_14_ with multiple lithium sites, the fraction of fast moving Li^+^ ions,^109^ and complete numerical simulations of the ^7^Li NMR line shape. As the temperature is increased even further (>500 K), Li_3_P_5_O_15_ is in the fast motional regime, resulting in averaging of the dipolar interaction through increased mobility, giving rise to narrow spectra with line widths of ∼750 Hz. The Li^+^ jump rate, τ^–1^, is on the order of the ^7^Li central transition NMR line width in the rigid lattice regime and quantified at the temperature of the inflection point, yielding a value of 2.8 × 10^4^ s^–1^ at 424 K comparable to values obtained for La_3_Li_3_W_2_O_12_^110^ and Li_4_SiO_4_.^111^

The NMR and AC impedance spectroscopy activation energies are cautiously comparable given the largely different approaches used that could potentially lead to contrasting values as observed for a range of different mobile ionic species,^112−114^ the validity of the Waugh and Fedin expression, the measurement uncertainty in the experimentally reported activation barriers based on AC impedance spectroscopy,^115^ and the possibility of multiple available pathways for Li^+^ hopping processes (see BVS data below).

To evaluate preliminarily the capability of Li_3_P_5_O_14_ as a solid electrolyte for Li plating and stripping, a symmetric Li|Li_3_P_5_O_14_|Li cell was prepared with a Li_3_P_5_O_14_ layer thickness of approximately 300 μm and cycled galvanostatically at 298 K with a current density of ±2.5 μA·cm^–2^. A steady test over 100 h with minimal changes in the stripping/plating overpotentials was observed (Figure 11g). This is indicative of a reasonably stable Li_3_P_5_O_14_|Li^0^ interface at room temperature. The interfacial reaction with Li is thus considered to be suppressed kinetically. The AC and DC interfacial resistivities are estimated by following a reported procedure (as described in the Supporting Information) and summarized in Table S13.^116^ The interfacial resistivity (two Li_3_P_5_O_14_|Li interfaces) accounts for about 79% and 37% of the total resistivity of the Li|Li_3_P_5_O_14_|Li cells based on the AC and DC data, respectively. Such a high interfacial resistivity suggests the formation of a passivation layer between the Li_3_P_5_O_14_ and Li metal, consistent with the theoretical computation of thermodynamic instability to lithium and the observed kinetically stable interface in the plating/stripping experiments. Given the magnitude of ionic conductivity of Li_3_P_5_O_14_ and large interfacial resistivity at room temperature, large overpotentials and ohmic losses are expected to drive plating/stripping processes, even at very low current densities. Higher temperatures have been used to improve Li_3_P_5_O_14_|Li interfacial contact and enhance the bulk ionic conductivity of the solid electrolyte. However, preliminary investigations suggest that the interfacial resistance grows significantly at higher temperatures (i.e., 323 K, see Figure S14). Therein, the high temperature may facilitate rapid decomposition reactions at the interface of Li_3_P_5_O_14_|Li and such a passivation layer impedes the Li^+^ transport.

**Figure 12:**
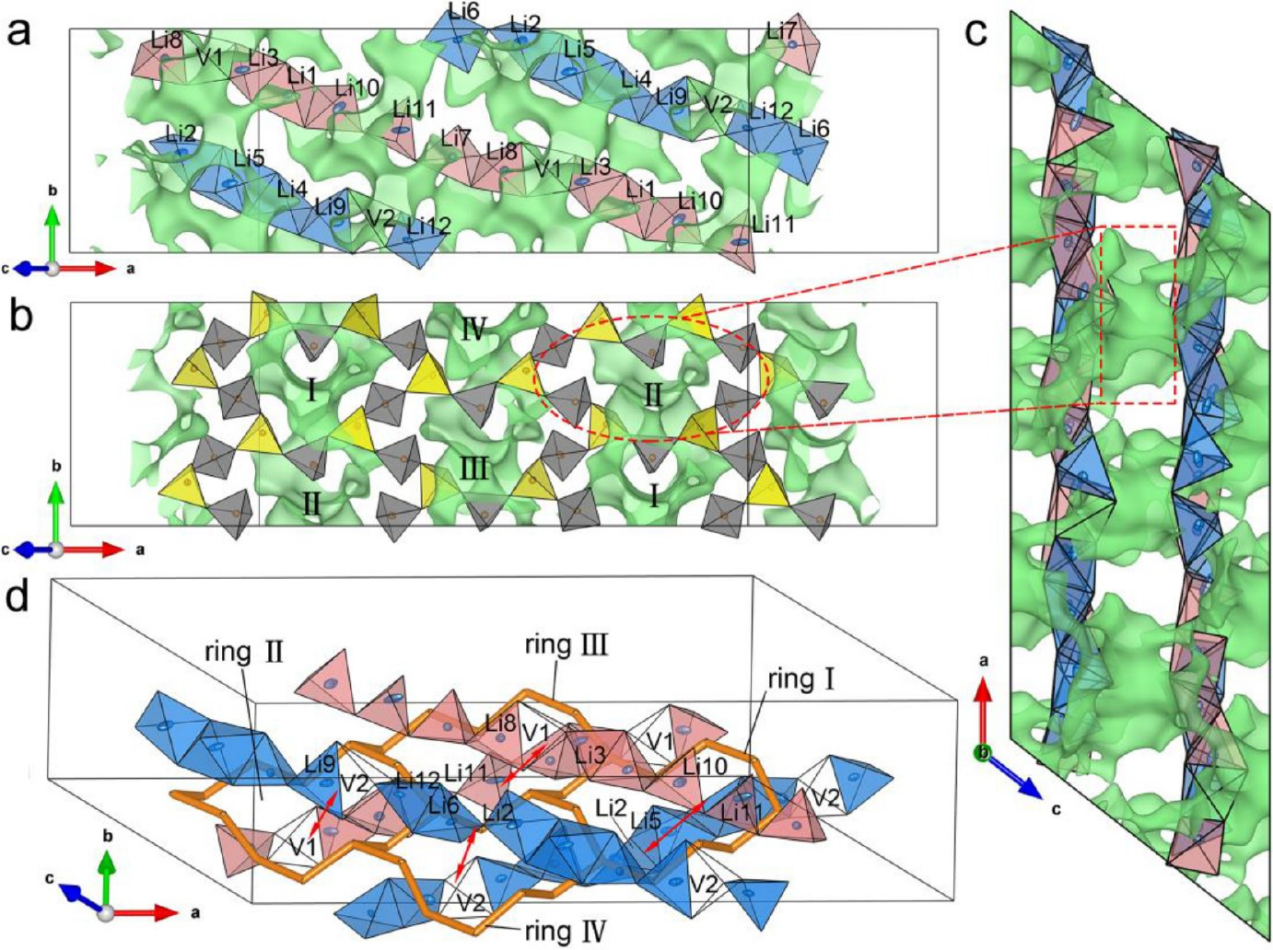
Potential Li migration pathways in Li_3_P_5_O_14_: (a) BVS map in the Li polyhedral layer (layer b), highlighting the intra-layer Li diffusion pathways. (b and c) BVS maps showing the Li diffusion pathways of inter-layer Li migration. (d) Inter-layer Li migration pathways to highlight the potential local jumps pass through four distinct 12-membered rings based on BVS maps in (b) and (c). Red arrows show the potential local jumps of inter-layer migration pathway. O atoms in PO_4_^3–^ tetrahedra are omitted to highlight the 12-membered P_12_O_36_^12–^ rings (orange). Isosurfaces (green) at a Li BVS of 1.0 valence unit are used to highlight potential pathways for Li ion conduction. The Li polyhedra in type A Li_6_O_16_^26–^ chain are colored in red, and those in type B Li_6_O_16_^26–^ are colored in blue. White tetrahedra show the interstitial sites between each finite Li_6_O_16_^26–^ chain. Internal and branching PO_4_^3–^ tetrahedra are colored in gray and yellow, respectively. Thermal ellipsoids are drawn with a 50% probability.

To understand the Li^+^ conduction mechanism in Li_3_P_5_O_14_, the crystal structure has been analyzed with a BVS map^68^ that shows a 3D isosurface connectivity displaying inter-layer and intra-layer migration pathways. The Li ion transport in the Li polyhedral layer in the *ab* plane is termed intra-layer migration, while the Li ion transport between two adjacent Li polyhedral layers (*c*-direction connectivity) is termed inter-layer migration. As shown in Figure 12a, the ordered Li_6_O_16_^26–^ chains along with the vacant tetrahedral sites form a possible intra-layer lithium diffusion pathway in the Li polyhedral layer. The intra-layer migration could occur either by a hopping mechanism of Li ions along the Li_6_O_16_^26–^ chains or by hopping between the two types of Li_6_O_16_^26–^ chains, where the local jumps between the two types of Li_6_O_16_^26–^ chains involved are between V1 and Li2 tetrahedral sites, between V1 and Li5 distorted square pyramid sites, between V2 and Li10 tetrahedral sites, and between V2 and Li11 tetrahedral sites.

BVS maps in Figures 12b and 12c highlight the potential inter-layer lithium migration pathways, where the 12-membered P_12_O_36_^12–^ rings in the P_20_O_56_^12–^ layers provide a window that mobile Li ions can traverse. Based on BVS mapping, the potential local jumps pass through these 12-membered rings are highlighted in Figure 12d, indicating that vacant tetrahedral sites, V1 or V2, are involved with adjacent Li occupied sites in the adjacent Li polyhedral layer for inter-layer migration with rings II–IV, while only Li occupied sites were involved for ring I migration. The local jumps of inter-layer migration with ring I are between type A and B Li_6_O_16_^26–^ chains involving Li5 square pyramid sites, and Li10, Li11 tetrahedral sites. Similarly, for inter-layer migration with ring II, the local jumps are between type A and B Li_6_O_16_^26–^ chains involving V1, Li3, Li8, V2, Li9, and Li12 tetrahedral sites. Those for ring III are between two of type A Li_6_O_16_^26–^ chains involving V1, Li3, Li8, Li10, Li11 tetrahedral sites. Those for ring IV are between two of type B Li_6_O_16_^26–^ chains involving Li2, Li6, V2, Li9, and Li12 tetrahedral sites. The vacant tetrahedral sites in Li polyhedral layers and 12-membered rings in ultraphosphate layers increase the probability of Li ions migrating through intra-layer and inter-layer migration pathways, which are most likely responsible for the higher ion conductivity than those of other ternary lithium phosphates and conventional coating materials. The structural features of Li_3_P_5_O_14_ are consistent with the criteria of crystal structures identified as enabling lithium-ion conduction using topological analysis and ab initio molecular dynamics simulations.^117^

**Figure 13:**
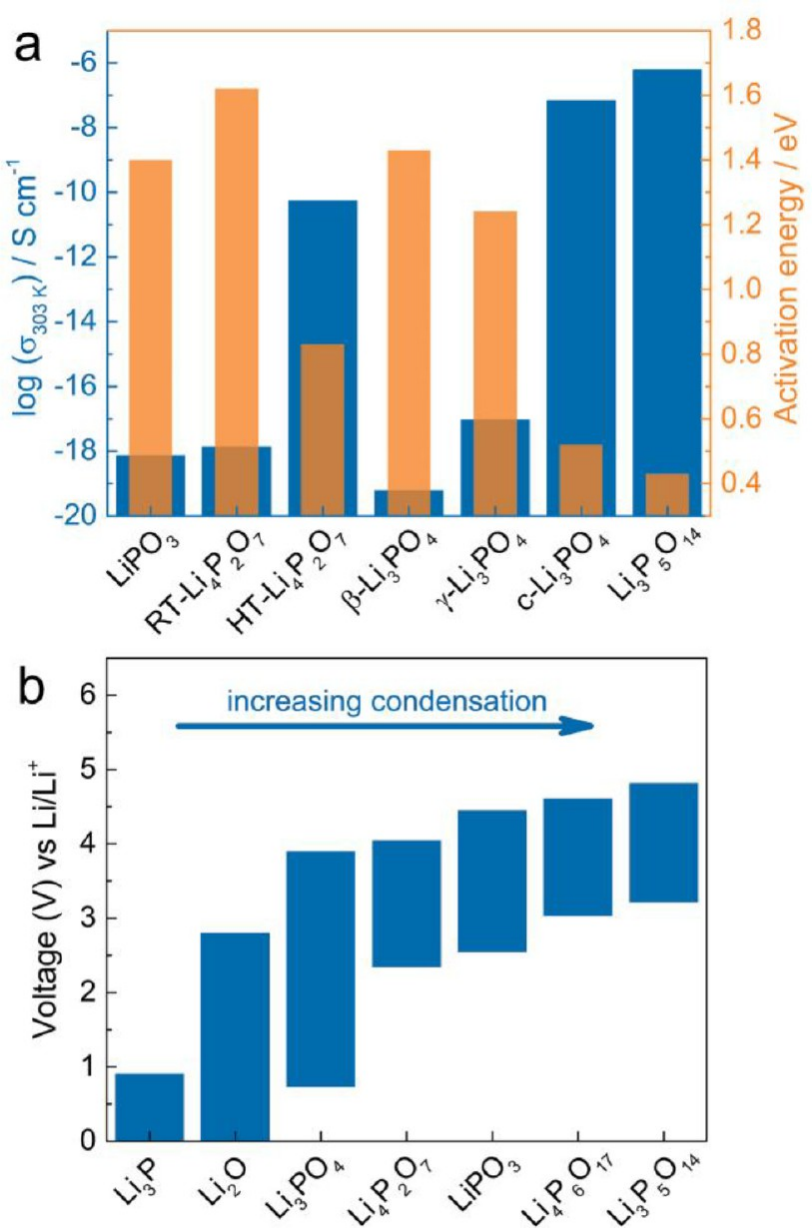
(a) Comparison of total ionic conductivity (303 K) and activation energy for materials in the Li–P–O phase field. (b) Calculated theoretical thermodynamic voltage stability windows of compounds in Li–P–O phase field.

It is instructive to compare the ionic conductivity and activation energy to other ternary oxides in the Li–P–O phase field, where there are 11 reported crystalline phases. There are three polymorphs of lithium orthophosphate Li_3_PO_4_, five polymorphs of the pyrophosphate Li_4_P_2_O_7_, and three polymorphs of the polyphosphate LiPO_3_. As summarized in Table S10, apart from a polymorph of Li_3_PO_4_ with a face-centered cubic structure (*c*-Li_3_PO_4_), all other reported phases in this system have very limited ionic conductivity at room temperature (∼10^–20^–10^–11^ S cm^–1^). *c*-Li_3_PO_4_, prepared by heat treatment in a flowing humid hydrogen atmosphere, has a relatively high total conductivity (6.9 × 10^–8^ S cm^–1^ at 303 K) compared with those other ternaries in the Li–P–O phase field. It should be noted that *c*-Li_3_PO_4_ is also a solid protonic conductor with total ion and proton conductivity at 873 K of 2.4 × 10^–2^ and 6 × 10^–3^ S cm^–1^, respectively.^118^ According to the literature, due to difficulties in accurately measuring protonic conductivities at low temperatures for *c*-Li_3_PO_4_, the activation energy for the proton conductivity (0.21 eV) is unreliable. Therefore, there is no available room temperature value for the Li ion conductivity of *c*-Li_3_PO_4_. The room temperature ionic conductivities and activation energy of Li_3_P_5_O_14_ and some known lithium phosphates in the Li–P–O phase field are highlighted in Figure 13a. The lithium stochiometric content (Li/P ratio) decreases in the sequence ortho-, poly-, cyclo-, and ultraphosphates, and Li_3_P_5_O_14_ has the lowest ratio of Li/P among all ternary phases in the Li–P–O field. However, Li_3_P_5_O_14_ has the highest ionic conductivity and is at least 1 order of magnitude higher than *c*-Li_3_PO_4_ and 4 orders of magnitude higher than other materials (Table S10), which can be attributed to its unique 3D lithium migration pathways.

The electrochemical stability window of the two new ultraphosphates was calculated with an approach analogous to the Li-grand canonical phase diagram and compared with several representative Li–P–O materials. Interestingly both phases appear to have a relatively high thermodynamic stability against oxidation (Figure 13b), in particular Li_3_P_5_O_14_, which is predicted to be stable up to 4.8 V, which is higher than other solid electrolytes, including the other lithium phosphates and the LiPON thin-film electrolytes, and than conventional coatings materials such as Li_4_Ti_5_O_12_, Li_2_ZrO_3_, LiTaO_3_, LiNbO_3_, LiAlO_2_, and Li_2_SiO_3_.^69,70,72^ However, the stability against reduction by the Li metal is relatively poor (a lower limit of the electrochemical window of 3.4 V) compared with other lithium phosphates. It should be noted that the theoretical computation provides an estimation of the worst-case scenario of the stability limit of solid electrolytes, as the reactions with the cathode and anode are often kinetically limited.^71^

For lithium phosphates, the thermodynamic stability against oxidation increases as the degree of phosphate condensation increases, while the stability against reduction decreases. The degree of phosphate condensation decreases in the order Li_3_P_5_O_14_ > Li_4_P_6_O_17_ > LiPO_3_ > Li_4_P_2_O_7_ > Li_3_PO_4_. For Li_3_P_5_O_14_, where the P_20_O_56_^12–^ anionic layers have the highest degree of condensation, the thermodynamic stability against oxidation is highest while the stability against reduction is poorest. Li_4_P_6_O_17_ has the second highest degree of anionic condensation; hence, the stability against oxidation is second only to Li_3_P_5_O_14_ and the stability against reduction is only higher than that of Li_3_P_5_O_14_. Lithium orthophosphate Li_3_PO_4_ has the lowest degree of anionic condensation, with isolated PO_4_^3–^ tetrahedra, and thus the poorest stability against oxidation and the highest stability against reduction.

This regular progression can be explained by comparing the possible anodic and cathodic reactions in these different lithium phosphates. The computed anodic reaction of Li_3_PO_4_ above the 3.9 V stability limit is 4Li_3_PO_4_ → 4Li + 2Li_4_P_2_O_7_ + O_2_, indicating that the reaction is aided by the presence of a product with a higher degree of phosphate condensation than the reactant. For the Li_3_P_5_O_14_ ultraphosphate with the highest degree of anionic condensation among known lithium phosphates, the computed reaction is 4Li_3_P_5_O_14_ → 12Li + 10P_2_O_5_ + 3O_2_ and requires a greater driving force due to the absence of a stable ternary product with a higher degree of phosphate condensation. Moreover, as noted by Ceder et al. the phosphate condensation decreases the P/O ratio and enhances the hybridization of each O with P, thereby increasing the oxidation stability in the sequence orthophosphate, pyrophosphate, and polyphosphate.^58,119^ The ultraphosphates extend this sequence. It has been proposed though that there is a trade-off between high ionic conductivity and high electrochemical stability to oxidation, as generally good ionic conductivity requires high Li content for polyanionic oxides, which was observed to be negatively correlated to oxidation stability.^58,120^ In comparison with other reported ternaries in the Li–P–O phase field, Li_3_P_5_O_14_ achieves both improved Li-ion mobility and a higher thermodynamic stability to oxidation with a lower Li content.

The computed cathodic reaction of Li_3_PO_4_ at its stability limits is 8Li + Li_3_PO_4_ → 4Li_2_O + Li_3_P. Li_3_PO_4_ has the lowest degree of phosphate condensation and thus decomposes into these two binary phases. The other phosphates can all decompose into another lithium phosphate with a lower level of condensation. For example, Li_3_P_5_O_14_ decomposes at voltages below 3.24 V in the reaction 17Li_3_P_5_O_14_ + 5Li → 14Li_4_P_6_O_17_ + P. It would be expected that the reaction at reducing potentials would proceed sequentially through the products Li_4_P_6_O_17_ (3.2 V), LiPO_3_ (3.0 V), Li_4_P_2_O_7_ (2.5 V), and Li_3_PO_4_ (2.3 V) until finally below 0.7 V affording Li_2_O and Li_3_P (essentially tracking the bottom of the bars in Figure 13b). This could explain kinetic stability at low voltage, and also could result in the formation of a stable interface between Li_3_P_5_O_14_ and Li metal. In all cases, kinetic stability of interfaces that are thermodynamically unstable is possible.

## 4 Conclusion

Reported lithium phosphates are based on isolated, terminal, or internal phosphate tetrahedra that share zero, one, or two of their oxygens to form extended structural units. The lithium ultraphosphates Li_3_P_5_O_14_ and Li_4_P_6_O_17_ introduce a distinct fundamental structure-building motif, the branching phosphate tetrahedron that shares three oxygens with other tetrahedra. This generates ultraphosphate anions with extended structural motifs distinct from those in previously reported Li phosphates, because of the different combinations of tetrahedral connectivity that arise when the branching tetrahedra are included. The combination of 2- and 3-connectivity in the infinite P_20_O_56_^12–^ anion in Li_3_P_5_O_14_ creates 12-membered rings that generate three-dimensional transport pathways affording higher Li conductivity than other lithium phosphates, and comparable to that of materials used as thin film electrolytes, despite the higher condensation of the network and reduced Li content in the new materials. The higher phosphate condensation introduced by the branching phosphates in the new structures reported here combines this higher conductivity with higher intrinsic stability to electrochemical oxidation than previous phosphates, and indeed all other solid electrolytes evaluated computationally. With a conductivity matching that required for cathode coatings, and a kinetically stable interface to lithium at room temperature, Li_3_P_5_O_14_ illustrates the opportunities offered by new structural motifs for enhanced Li transport in phosphates, which benefit from inexpensive components and ease of handling. The control of defect chemistry through substitution into these new ultraphosphate structures requires investigation to optimize the identified transport and stability properties.

## Supporting Information Available

Structural information on Li_3_P_5_O_14_ and Li_4_P_6_O_17_ (refinement details, atomic positions, bond distances and angles), PXRD patterns, Pawley refinement, SEM/TEM images (Li_3_P_5_O_14_), TGA–DTA, AC impedance, DC polarization, EIS Nyquist plots of Li|Li_3_P_5_O_14_|Li cell, crystallographic data for Li_3_P_5_O_14_ (CCDC 2096561 for synchrotron data, CCDC 2096562 for lab data) and Li_4_P_6_O_17_ (CCDC 2096563) (PDF)

## Accession Codes

CCDC 2096561–2096563 contain the supplementary crystallographic data for this paper. These data can be obtained free of charge via www.ccdc.cam.ac.uk/data_request/cif, or by emailing data_request@ccdc.cam.ac.uk, or by contacting The Cambridge Crystallographic Data Centre, 12 Union Road, Cambridge CB2 1EZ, UK; fax: +44 1223 336033.
