# Supplementary material for: Extended Condensed Ultraphosphate Frameworks with Monovalent Ions Combine Lithium Mobility with High Computed Electrochemical Stability
Source: J Am Chem Soc. 2021 Oct 22;143(43):18216–32. doi: 10.1021/jacs.1c07874 (PMC8569803; doi:10.1021/jacs.1c07874)
Supplement: Supplementary file 1 — ja1c07874_si_001.pdf [file ja1c07874_si_001.pdf]

Supporting Information for "Extended Condensed Ultraphosphate Frameworks with Monovalent Ions Combine Lithium Mobility with High Computed Electrochemical Stability"

Guopeng Han[1], Andrij Vasylenko[1], Alex R. Neale[1,2], Benjamin B. Duff[1,2], Ruiyong Chen[1], Matthew S. Dyer[1], Yun Dang[1], Luke M. Daniels[1], Marco Zanella[1], Craig M. Robertson[1], Laurence J. Kershaw Cook[1], Anna-Lena Hansen[3], Michael Knapp[3], Laurence J. Hardwick[1,2], Frédéric Blanc[1,2], John B. Claridge[1], and Matthew J. Rosseinsky[1,*]

[1] Department of Chemistry, University of Liverpool, Crown Street, Liverpool, L69 7ZD, United Kingdom

[2] Stephenson Institute for Renewable Energy, University of Liverpool, Peach Street L69 7ZF Liverpool, United Kingdom

[3] Institute for Applied Materials - Energy Storage Systems, Karlsruhe Institute of Technology, Hermann-von-Helmholtz-Platz 1, 76344 Eggenstein-Leopoldshafen, Germany

* Corresponding author: rossein@liverpool.ac.uk

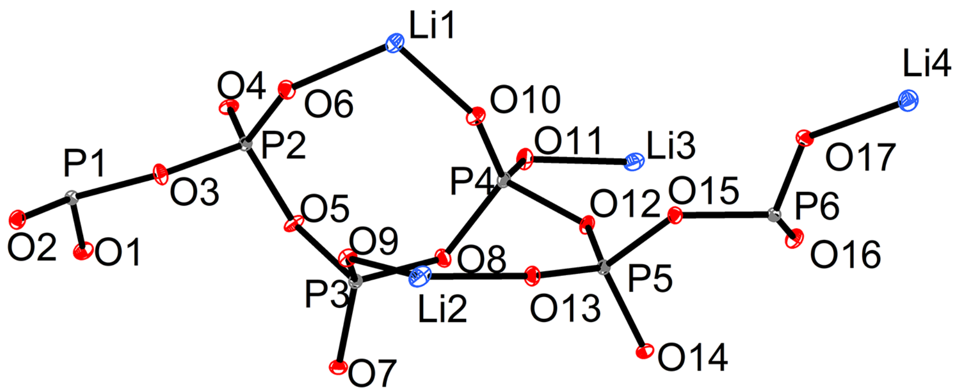

Figure S1. The asymmetric unit of $Li_4P_6O_{17}$. Displacement ellipsoids are drawn at 50% probability level. All the atoms occupy 2i sites and are ordered.

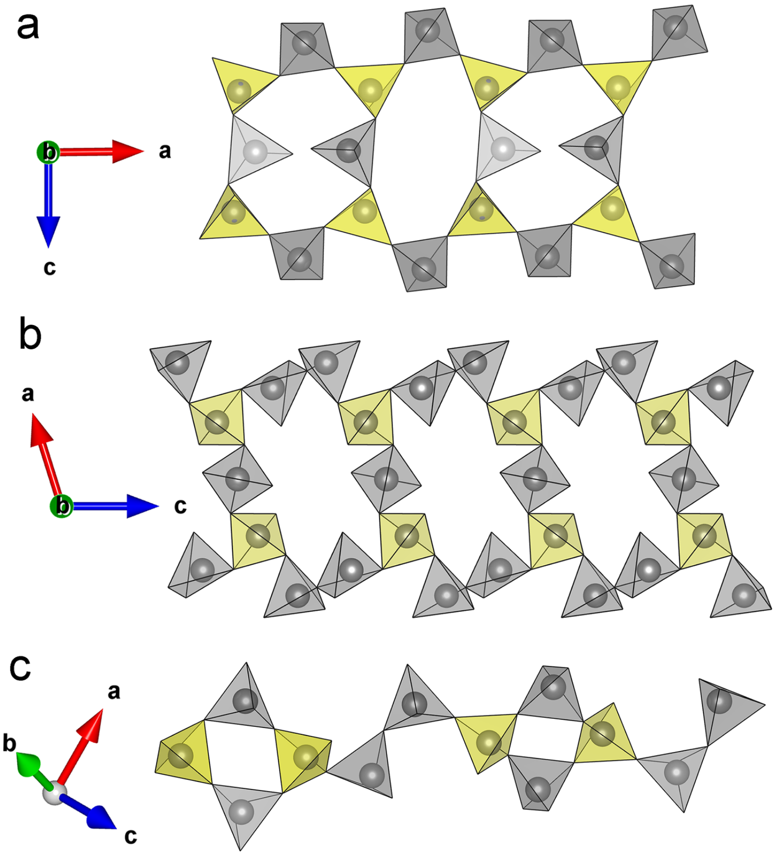

Figure S2. Comparison of 1D ultraphosphate ribbons in **a)** monoclinic LaP_5O_{14}, **b)** $CaYP_7O_{20}$ and **c)** $Li_4P_6O_{17}$. Internal PO_4^{3-} tetrahedra are colored in grey, branching PO_4^{3-} tetrahedra are colored in yellow.

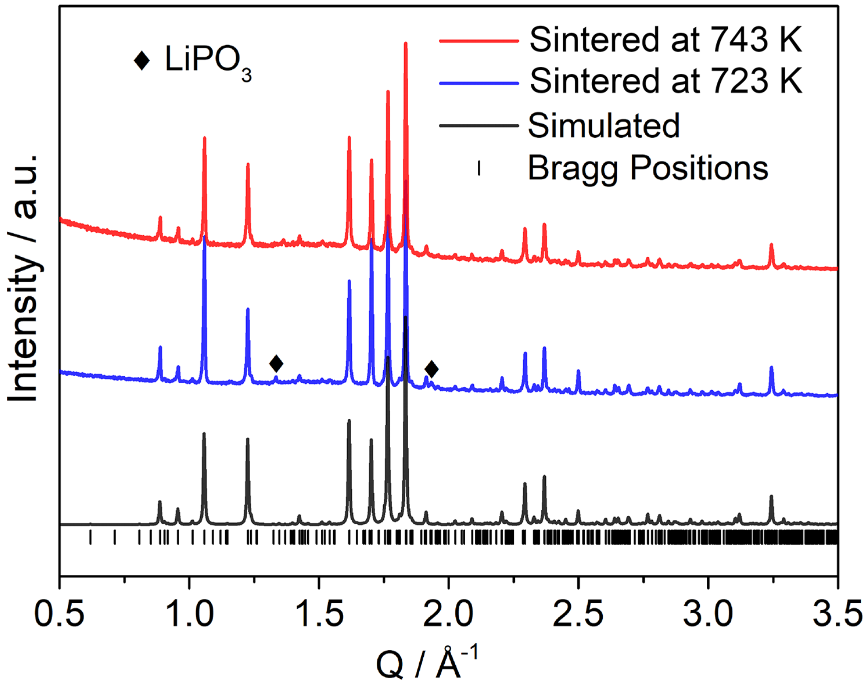

Figure S3. PXRD patterns for $Li_3P_5O_{14}$. Blue XRD pattern show the powder sample sintered at 723 K, the sample was then pelleted and subjected to a second heat treatment up to 743 K for 24 h leading to a single phase powder (red pattern). Presence of $LiPO_3$ is denoted with diamond symbols.

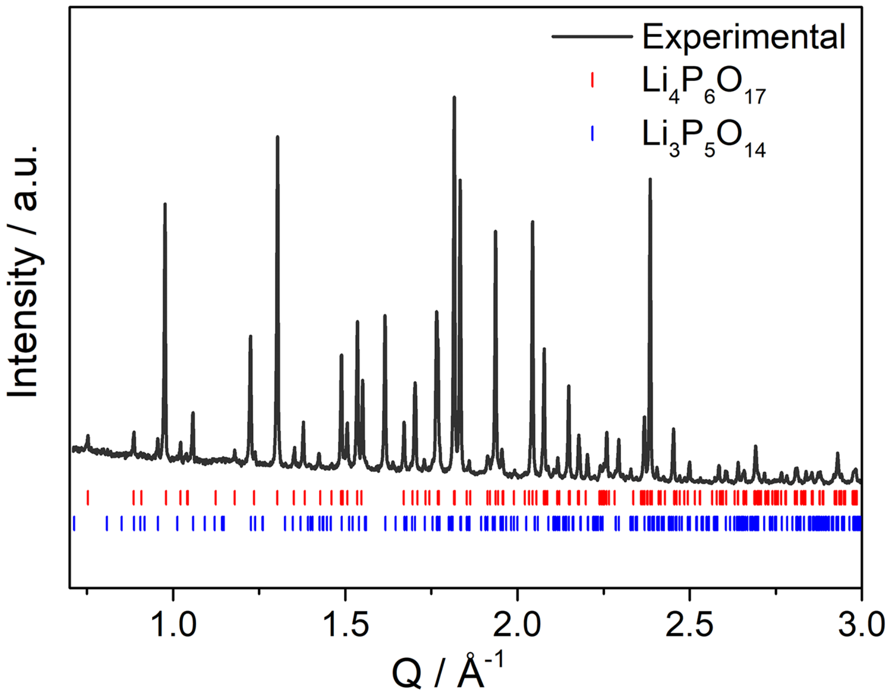

Figure S4. PXRD pattern of sample which yielded $Li_4P_6O_{17}$ and $Li_3P_5O_{14}$ crystals. $Li_4P_6O_{17}$ have not been isolated after trying various synthesis conditions such as temperature and time with stoichiometric ratio of Li_2O and P_2O_5. Red and blue tick marks represent positions of Bragg reflections for $Li_4P_6O_{17}$ and $Li_3P_5O_{14}$ respectively.

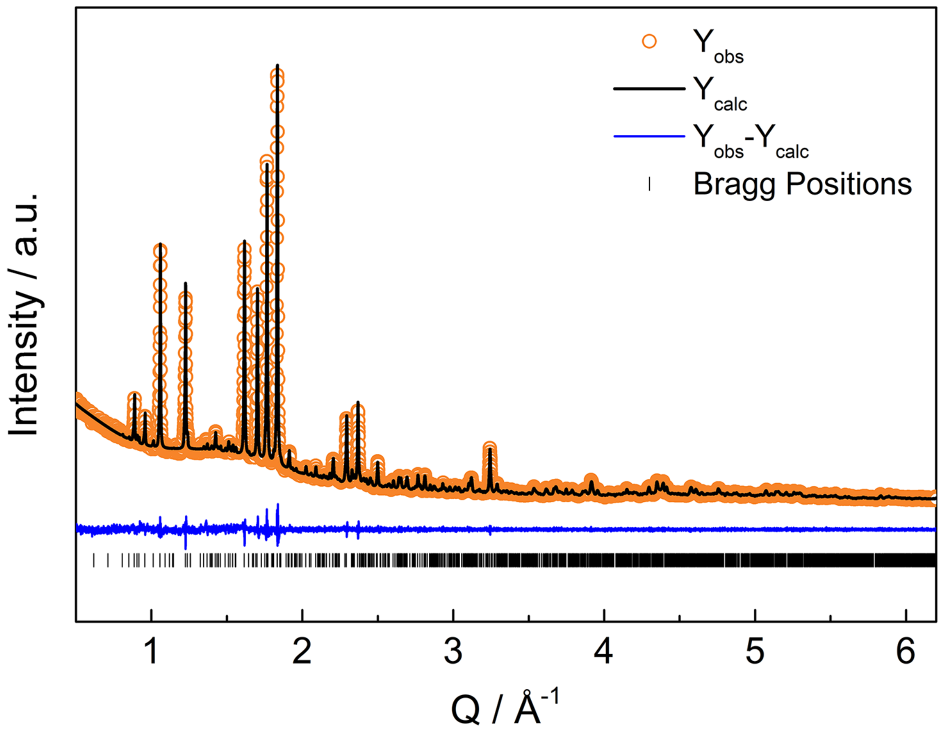

Figure S5. Pawley fit to the laboratory powder pattern of $Li_3P_5O_{14}$ using the lattice parameters and space group determined from single crystal diffraction of with Y_{obs} (orange dots), Y_{calc} (black line), Y_{obs}–Y_{calc} (blue line), and Bragg reflections (black tick marks). R_{wp}= 4.04%, R_{exp} = 3.16%, R_p = 3.05%, χ^2 = 1.64.

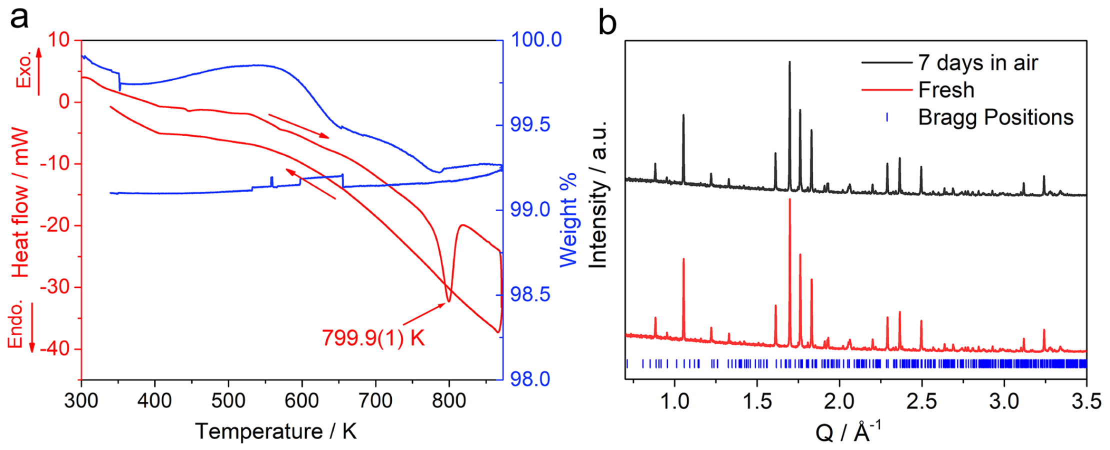

Figure S6. **a)** TGA–DTA curve of $Li_3P_5O_{14}$. The endothermic peak at 799.9(1) K in the heat flow trace is due to melting. **b)** Powder XRD patterns of fresh pellet of $Li_3P_5O_{14}$ (red pattern) and after exposure to air for 7 days (black pattern) highlighting the stability of $Li_3P_5O_{14}$.

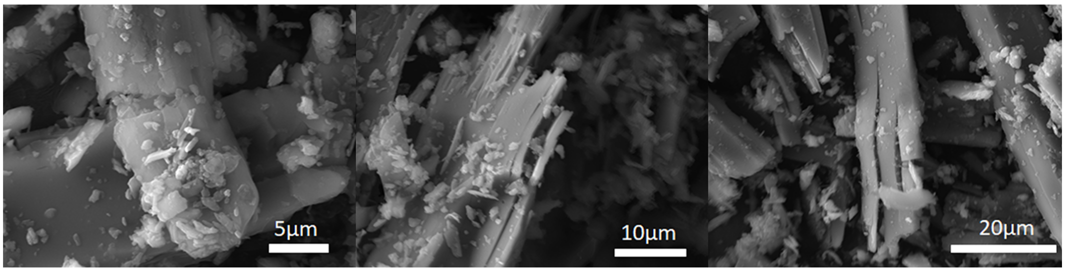

Figure S7. SEM images of flattened rod-like particles.

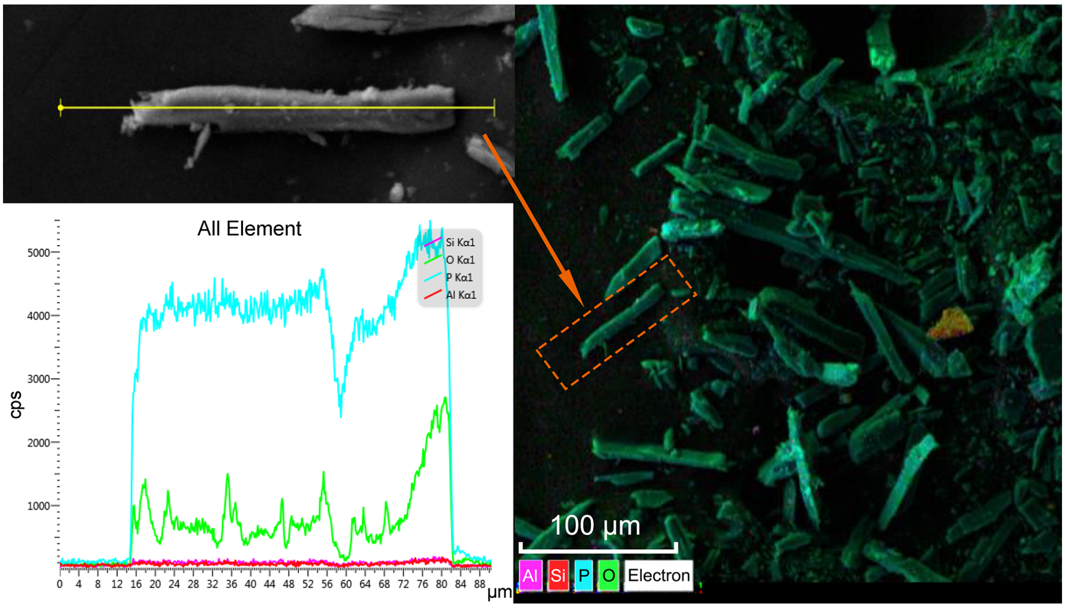

Figure S8. SEM–EDX elemental mapping of a sample of rod like particles. the composite image is shown on the right while the SEM linear EDX map of a rod like particle is shown on the left. P and O signal are plotted in light blue and green, respectively. Si and Al is plotted in pink and red, respectively. Rod like structures contain P and O. Si and Al impurities are present in the samples and seem to be concentrated into small particles likely introduced from the synthesis container.

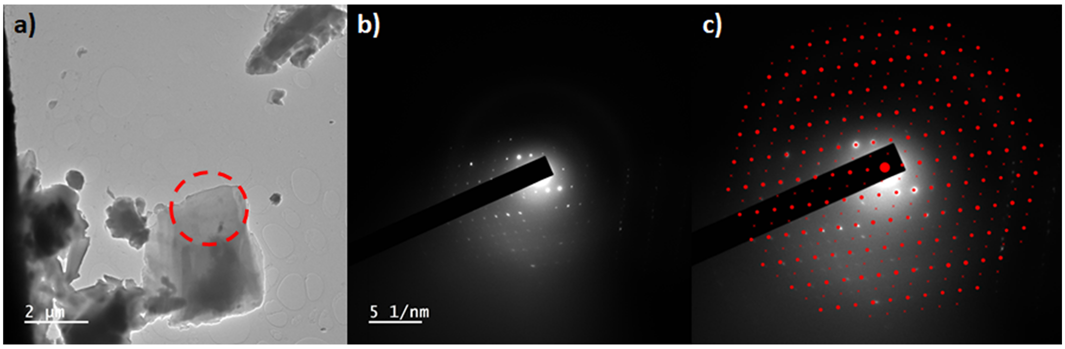

Figure S9. **a)** TEM image of fragment of rod like particle. The area selected for electron diffraction is highlighted in red. **b)** Selected area electron diffraction image. **c)** Comparison between the observed and simulated electron diffraction pattern viewed along the [123] zone axis.

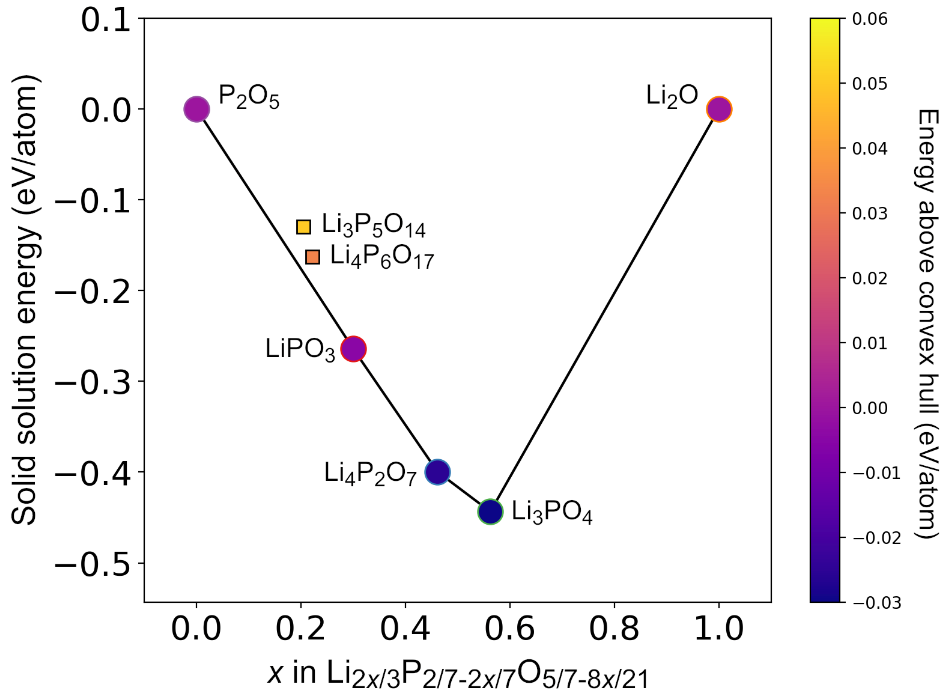

Figure S10. Formation energy of the compositions as a solid solution between P_2O_5 and Li_2O calculated with the meta-generalized gradient approximation with strongly correlated and appropriated normed (meta-GGA SCAN) functional.[1] The values of the total enthalpies for $Li_3P_5O_{14}$ and $Li_4P_6O_{17}$, calculated with meta-GGA SCAN are underestimated, resulting in the formation energies of these thermodynamically stable compositions to be slightly above the convex hull.

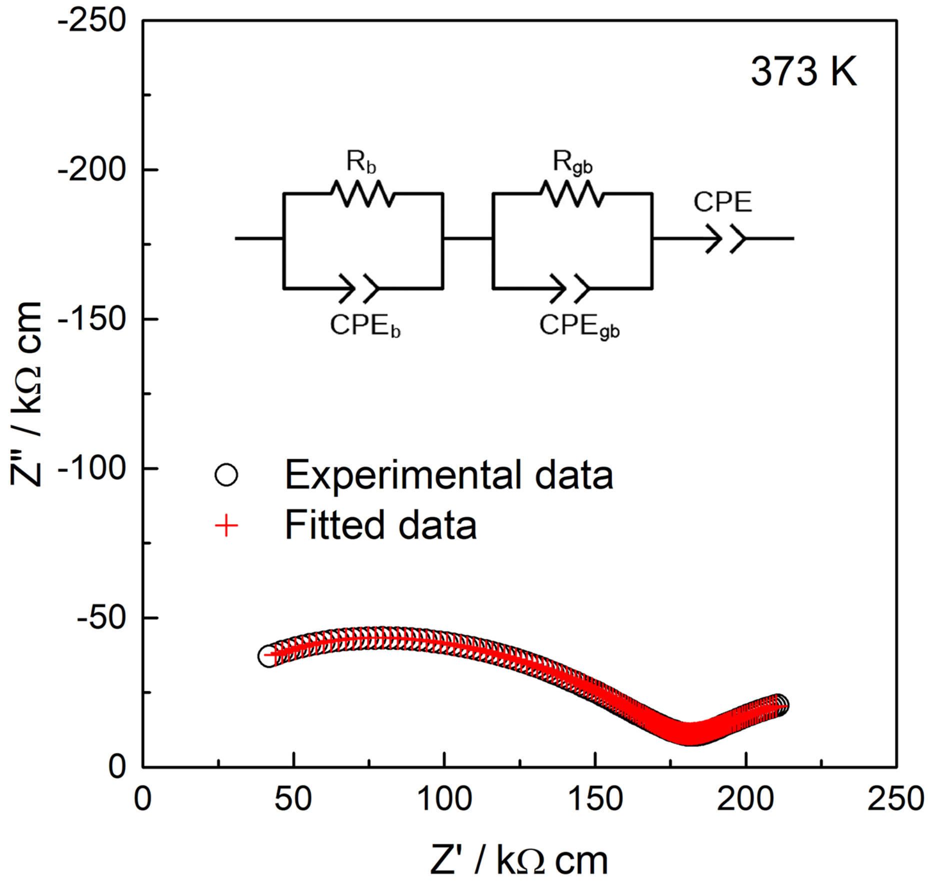

Figure S11. Nyquist plot at 373 K obtained from a Au|$Li_3P_5O_{14}$|Au cell, showing a high–frequency arc and a low-frequency inclined spike. Impedance is normalized for geometry. The inset is the equivalent circuit used to model the data, *R* – Resistance, *CPE* – Constant Phase Element. The low-frequency spike is attributed to the electrode double–layer phenomenon.

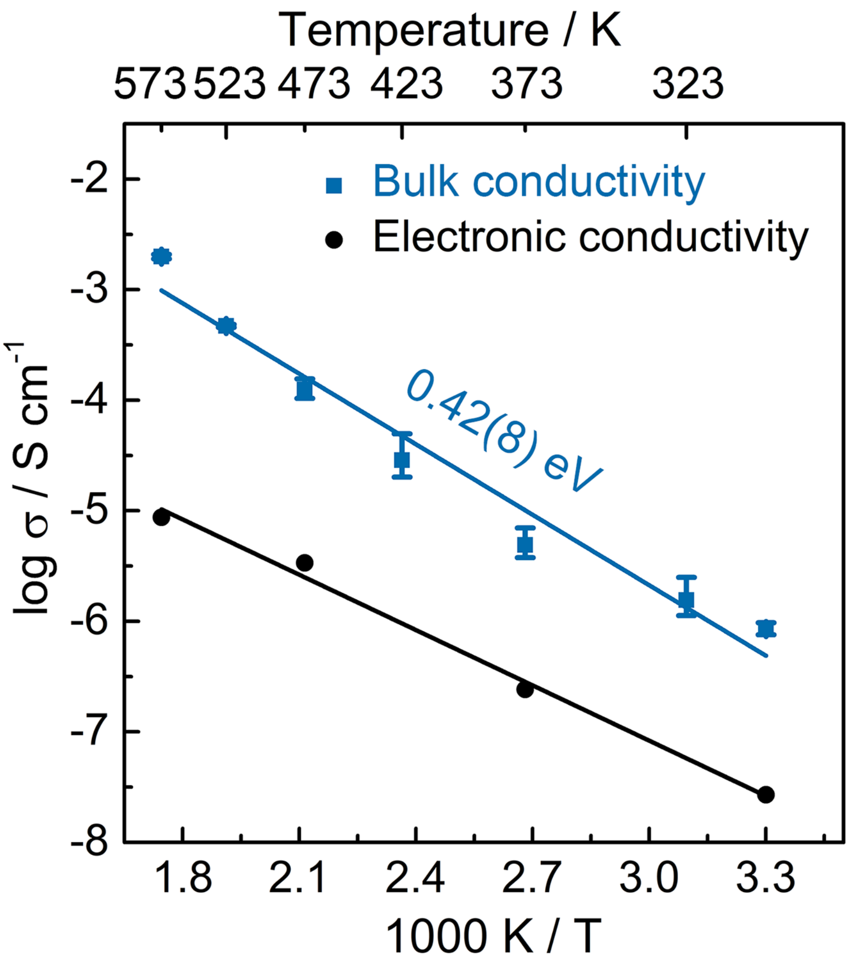

Figure S12. Arrhenius plots of the bulk conductivity measured by AC impedance spectroscopy (blue circles) and electronic conductivity measured by DC polarization (black circles).

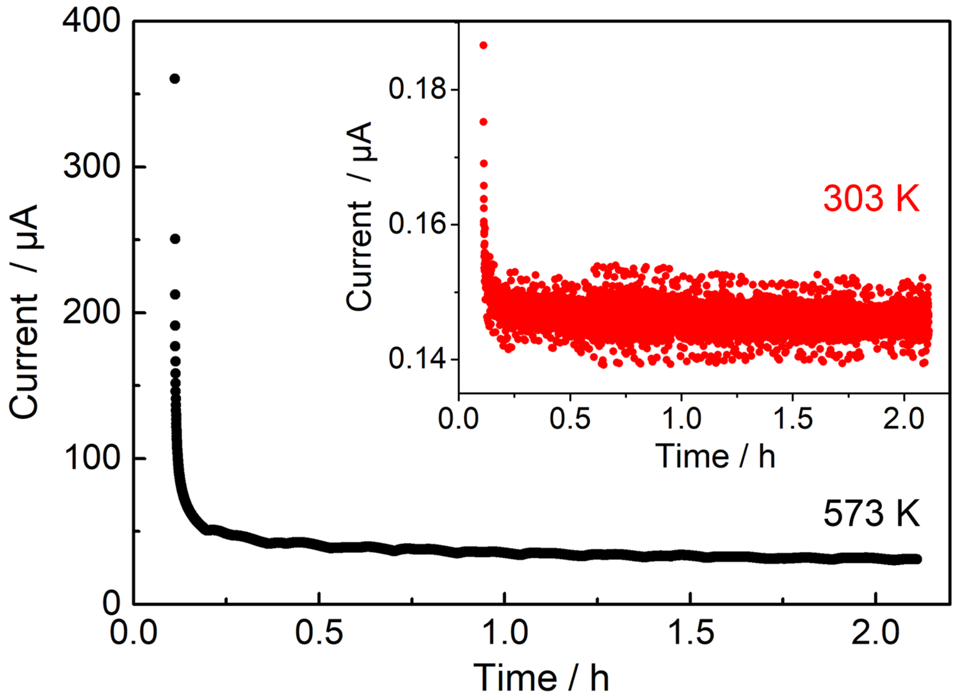

Figure S13. DC polarization measurement at 573 K (black) on a Au|$Li_3P_5O_{14}$|Au configuration with applied voltage 1 V. Inset shows data at 303 K (red).

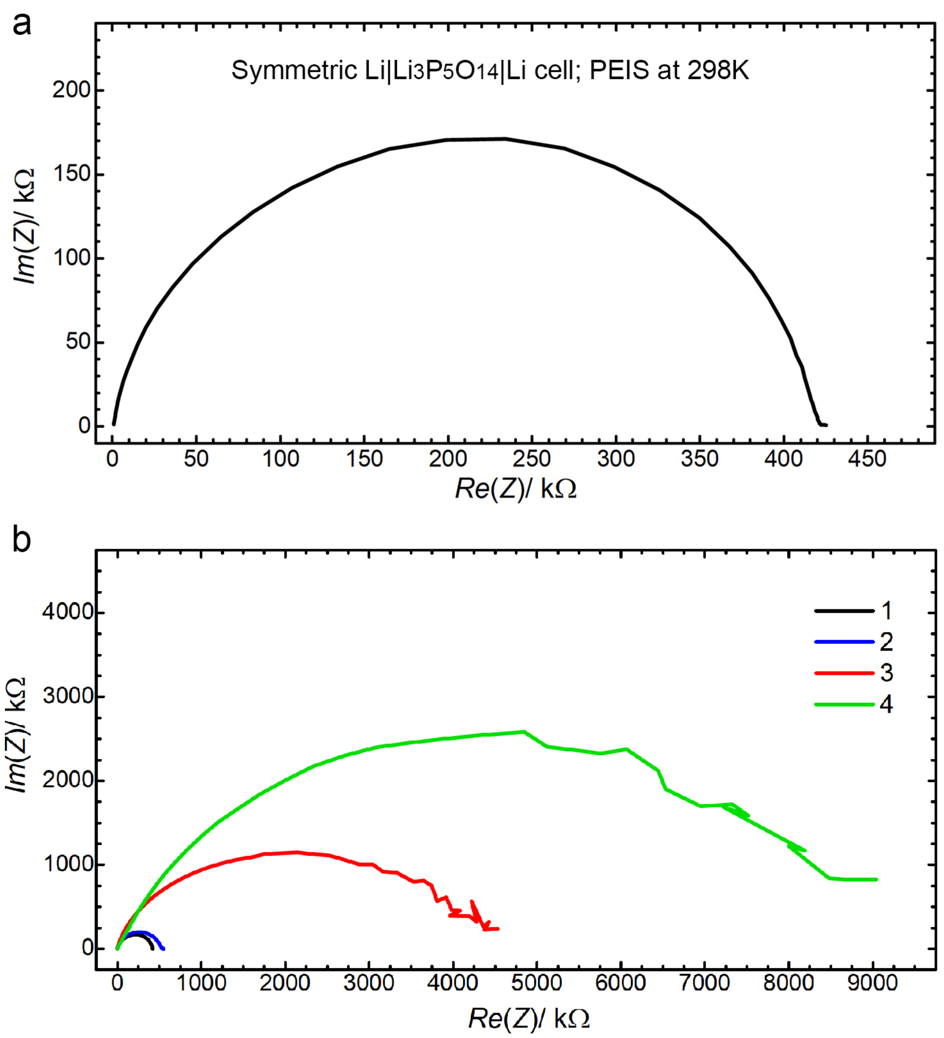

Figure S14. EIS Nyquist plots of a Li|$Li_3P_5O_{14}$|Li cell **a)** at 298K and **b)** as the temperature is increased from 298 K (curve 1) to 323 K (curve 4).

Table S1. Crystal data, data collection and structure refinement parameters of $Li_3P_5O_{14}$ and $Li_4P_6O_{17}$

Empirical formula	$Li_3P_5O_{14}$ Lab data	$Li_3P_5O_{14}$ Synchrotron	$Li_4P_6O_{17}$ Lab data
Formula weight	399.67	399.67	485.58
Temperature/K	293(2)	100.0	100
Crystal system, Space group	Monoclinic, *Cc*	Monoclinic, *Cc*	Triclinic, $P\bar{1}$
a/Å	33.3764(13)	33.2080(8)	7.3721(3)
b/Å	11.0005(2)	10.95400(10)	8.9291(3)
c/Å	15.0881(6)	14.9916(4)	10.8581(4)
α/°	90	90	79.596(3)
β/°	128.046(6)	127.857(4)	81.961(3)
γ/°	90	90	70.413(3)
Volume/Å^3	4362.6(4)	4305.7(2)	659.89(4)
Z	16	16	2
ρ_{calc}g/cm^3	2.434	2.466	2.444
μ/mm^{-1}	0.921	0.861	0.916
F(000)	3136	3136	476
Crystal size/mm^3	0.175 × 0.11 × 0.07	0.07 × 0.03 × 0.03	0.187 × 0.168 × 0.161
Radiation	MoKα (λ = 0.71073)	synchrotron (λ = 0.6889)	MoKα (λ = 0.71073)
2Θ range for data collection/°	3.098 to 55.75	3.012 to 58.95	3.828 to 56.548
Index ranges	-43 ≤ h ≤ 43, -14 ≤ k ≤ 14, -19 ≤ l ≤ 17	-47 ≤ h ≤ 47, -15 ≤ k ≤ 15, -21 ≤ l ≤ 21	-9 ≤ h ≤ 7, -11 ≤ k ≤ 11, -14 ≤ l ≤ 14
Reflections collected	48389	36219	15020
Independent reflections	9218 [R_{int} = 0.0497, R_{sigma} = 0.0388]	12819 [R_{int} = 0.0466, R_{sigma} = 0.0459]	3271 [R_{int} = 0.0287, R_{sigma} = 0.0223]
Data/restraints/parameters	9218/2/793	12819/2/793	3271/0/244
Goodness-of-fit on F^2	1.052	1.025	1.075
Final *R* indexes [I>=2σ (I)]	R_1 = 0.0378, wR_2 = 0.0835	R_1 = 0.0343, wR_2 = 0.0851	R_1 = 0.0269, wR_2 = 0.0720
Final *R* indexes [all data]	R_1 = 0.0455, wR_2 = 0.0868	R_1 = 0.0355, wR_2 = 0.0861	R_1 = 0.0293, wR_2 = 0.0735
Largest diff. peak/hole/ e Å^{-3}	0.41/-0.48	0.78/-0.58	0.44/-0.58
Flack parameter	0.06(4)	-0.03(3)	—

Table S2. Fractional atomic coordinates (× 10^4) and equivalent isotropic displacement parameters (Å^2 × 10^3) for $Li_3P_5O_{14}$.

Atoms	Wyck	*x*	*y*	*z*	*$U_{(eq)}$

P1	4a	7249.1(5)	7738.9(12)	5202.9(10)	13.4(3)
P2	4a	6334.9(5)	9157.6(12)	4613.4(11)	13.5(3)
P3	4a	6469.4(5)	10673.4(11)	6394.8(11)	14.1(3)
P4	4a	5365.0(5)	8043.4(11)	3695.5(11)	12.8(3)
P5	4a	5545.8(5)	12049.6(12)	5433.4(11)	17.0(3)
P6	4a	6324.1(5)	4532.2(12)	4304.4(11)	14.6(3)
P7	4a	7198.3(5)	3037.1(12)	5008.5(12)	15.3(3)
P8	4a	5420.6(5)	3167.0(12)	3519.0(12)	17.9(3)
P9	4a	6392.8(5)	5548.9(11)	6214.7(10)	14.1(3)
P10	4a	5520.4(5)	6941.7(12)	5680.7(11)	12.9(3)
P11	4a	4740.5(5)	10485.9(12)	5078.4(11)	15.9(3)
P12	4a	3861.6(5)	11938.8(12)	4368.9(11)	14.6(3)
P13	4a	2994.2(5)	10452.6(12)	3723.4(10)	14.4(3)
P14	4a	4700.8(5)	5281.1(12)	5125.0(11)	13.9(3)
P15	4a	3787.3(5)	6731.5(12)	4416.9(11)	14.1(3)
P16	4a	3085.0(5)	9419.1(11)	5653.9(11)	14.8(3)
P17	4a	3992.2(5)	8072.9(11)	6343.4(11)	14.5(3)
P18	4a	2834.3(5)	5444.5(12)	3489.9(11)	15.2(3)
P19	4a	2992.6(5)	4468.3(12)	5534.4(11)	14.3(3)
P20	4a	3872.3(5)	2980.6(11)	6229.9(11)	14.9(3)
O1	4a	7120.7(16)	7239(4)	4168(3)	32.4(10)
O2	4a	7534.2(14)	7016(3)	6248(3)	25.8(9)
O3	4a	6753.3(14)	8188(3)	5034(3)	29.8(9)
O4	4a	6118.2(15)	9716(4)	3547(3)	34.3(10)
O5	4a	6594.4(14)	10051(3)	5623(3)	22.2(8)
O6	4a	6819.8(14)	11717(3)	6935(3)	22.2(8)
O7	4a	5903.1(13)	11146(3)	5405(3)	23.5(8)
O8	4a	6437.8(14)	9763(3)	7054(3)	26.2(9)
O9	4a	5426.0(14)	13072(3)	4600(3)	24.9(9)
O10	4a	5759.0(15)	12463(4)	6557(3)	29.0(9)
O11	4a	5032.6(13)	11353(4)	4779(3)	29.6(9)
O12	4a	5950.7(11)	8420(3)	4675(3)	17.0(7)
O13	4a	5348.2(13)	7074(3)	3000(3)	22.7(8)
O14	4a	5835.7(13)	7811(3)	6591(3)	22.1(8)
O15	4a	5233.9(13)	7475(3)	4476(3)	21.7(8)
O16	4a	5055.8(13)	9151(3)	3189(3)	20.6(8)
O17	4a	6699.0(13)	6652(3)	6797(3)	21.1(8)
O18	4a	6297.9(14)	5507(3)	5028(3)	19.2(8)
O19	4a	5810.1(13)	5797(3)	5728(3)	18.9(8)
O20	4a	6535.5(14)	4349(3)	6726(3)	26.7(9)
O21	4a	6156.9(13)	5076(3)	3246(3)	21.9(8)
O22	4a	6028.7(12)	3382(3)	4202(3)	20.0(8)
O23	4a	6887.3(12)	4071(3)	5122(3)	19.4(8)
O24	4a	7538.7(14)	2431(3)	6114(3)	22.7(8)

O25	4a	6853.2(14)	2341(3)	3968(3)	25.3(9)
O26	4a	5300.3(15)	1992(3)	2958(3)	29.7(10)
O27	4a	5152.8(14)	4299(3)	2949(3)	29.9(9)
O28	4a	5081.2(13)	6437(3)	5666(3)	19.8(8)
O29	4a	4525.6(15)	5042(3)	3993(3)	29.7(9)
O30	4a	4937.3(13)	4324(3)	6003(3)	19.6(8)
O31	4a	4254.0(13)	5867(3)	5107(3)	21.8(8)
O32	4a	3612.4(14)	7108(3)	3310(3)	22.1(8)
O33	4a	2537.9(13)	4031(3)	5492(3)	21.1(8)
O34	4a	3389.7(12)	6003(3)	4439(3)	22.3(8)
O35	4a	4390.5(14)	9746(3)	4075(3)	23.7(9)
O36	4a	5111.7(14)	9957(3)	6208(3)	22.9(8)
O37	4a	4427.6(13)	11505(3)	5207(3)	20.5(8)
O38	4a	3715.9(14)	12528(3)	3352(3)	25.9(9)
O39	4a	3792.4(14)	12818(3)	5071(3)	26.1(9)
O40	4a	3563.4(13)	10753(3)	4181(3)	21.8(8)
O41	4a	2885.0(14)	9231(3)	3215(3)	21.2(8)
O42	4a	2660.7(13)	11507(3)	3131(3)	22.0(8)
O43	4a	3096.0(14)	10375(3)	4914(3)	20.6(8)
O44	4a	3395.8(12)	8284(3)	5771(3)	20.8(8)
O45	4a	3244.8(15)	9939(3)	6716(3)	26.3(9)
O46	4a	2531.4(12)	8920(3)	4832(3)	22.5(8)
O47	4a	4262.4(13)	9246(3)	6783(3)	20.7(8)
O48	4a	3932.6(14)	7841(3)	5206(3)	21.3(8)
O49	4a	4162.7(14)	6977(3)	7016(3)	24.7(9)
O50	4a	2464.7(13)	6422(3)	2866(3)	26.5(9)
O51	4a	2876.3(14)	4433(3)	2912(3)	27.5(9)
O52	4a	4187.3(13)	4078(3)	6761(3)	22.1(8)
O53	4a	2725.9(13)	4886(3)	4313(3)	29.5(9)
O54	4a	3300.8(14)	5385(3)	6393(3)	24.8(9)
O55	4a	3288.6(12)	3288(3)	5688(3)	22.1(8)
O56	4a	3996.7(15)	1827(3)	6820(3)	33.2(10)
Li1	4a	7448(4)	12070(8)	7236(8)	25(2)
Li2	4a	5852(4)	10627(10)	2236(8)	45(3)
Li3	4a	6926(4)	6829(9)	2794(8)	36(3)
Li4	4a	7379(3)	6959(7)	7287(8)	20.1(19)
Li5	4a	6678(5)	1916(8)	2569(8)	37(3)
Li6	4a	4958(4)	502(8)	2266(7)	21(2)
Li7	4a	4856(3)	5713(8)	2180(8)	22(2)
Li8	4a	5637(3)	13675(8)	7260(8)	24(2)
Li9	4a	3220(4)	11356(8)	7415(7)	31(2)
Li10	4a	3178(4)	6470(10)	7172(9)	42(3)
Li11	4a	4183(5)	5472(9)	7542(9)	47(3)
Li12	4a	4366(3)	544(8)	7787(7)	21(2)

*$U_{(eq)}$ is defined as 1/3 of the trace of the orthogonalized U_{ij} tensor (standard deviations in parentheses).

Table S3. Fractional atomic coordinates (× 10^4) and equivalent isotropic displacement parameters ($Å^2$ × 10^3) for $Li_4P_6O_{17}$.

Atoms	Wyck	*x*	*y*	*z*	*$U_{(eq)}$
P1	2i	12085.8(6)	1075.5(5)	4840.0(4)	5.15(10)
P2	2i	8017.8(6)	2190.9(5)	4278.1(4)	5.05(11)
P3	2i	7309.3(6)	4753.2(5)	2172.7(4)	5.06(11)
P4	2i	3367.6(6)	4644.6(5)	2030.7(4)	5.48(11)
P5	2i	1857.3(6)	8072.4(5)	1224.0(4)	4.90(10)
P6	2i	-2245.9(6)	9531.3(5)	841.0(4)	5.15(10)
O1	2i	12822.7(18)	500.4(15)	3618.6(12)	8.3(3)
O2	2i	13091.3(18)	1850.5(15)	5471.8(12)	8.2(3)
O3	2i	9876.0(18)	2260.4(15)	4779.4(12)	8.1(3)
O4	2i	8324.8(18)	400.2(15)	4166.2(12)	7.3(3)
O5	2i	8105.9(18)	2899.0(15)	2850.8(12)	8.0(3)
O6	2i	6289.9(18)	3011.2(15)	5037.9(12)	8.5(3)
O7	2i	8774.8(18)	4908.0(15)	1120.2(12)	7.4(3)
O8	2i	5491.6(18)	4735.1(15)	1548.8(12)	7.9(3)
O9	2i	6776.6(18)	5838.2(15)	3137.2(12)	7.5(3)
O10	2i	2901.8(19)	4763.3(16)	3375.1(12)	9.7(3)
O11	2i	3115.5(18)	3326.3(15)	1486.7(12)	8.8(3)
O12	2i	2087.1(18)	6279.1(15)	1198.8(12)	6.9(2)
O13	2i	3111.1(18)	8359.2(15)	2017.8(12)	8.5(3)
O14	2i	2151.6(18)	8693.6(15)	-222.0(12)	6.5(2)
O15	2i	-352.1(17)	8883.0(15)	1615.4(11)	6.6(2)
O16	2i	-1963.3(18)	8585.3(15)	-204.2(12)	8.1(3)
O17	2i	-3923.0(18)	9732.6(15)	1771.9(12)	8.2(3)
Li1	2i	3473(5)	4025(4)	5041(3)	10.7(6)
Li2	2i	5061(5)	8262(4)	3210(3)	12.2(6)
Li3	2i	1203(5)	3628(4)	379(3)	9.7(6)
Li4	2i	-6621(5)	11037(4)	1824(3)	11.0(6)

*$U_{(eq)}$ is defined as 1/3 of the trace of the orthogonalized U_{ij} tensor (standard deviations in parentheses).

Table S4. Anisotropic displacement parameters ($Å^2$ × 10^3) for $Li_3P_5O_{14}$. The anisotropic displacement factor exponent takes the form: $-2\pi^2[h^2a^{*2}U_{11} + 2hka^*b^*U_{12} + \ldots]$.

Atom	U_{11}	U_{22}	U_{33}	U_{23}	U_{13}	U_{12}
P1	14.2(7)	11.8(6)	16.4(7)	0.3(5)	10.5(6)	0.3(5)
P2	13.4(6)	12.7(6)	15.5(7)	-1.7(5)	9.4(6)	-0.8(5)
P3	15.1(7)	11.1(6)	15.8(7)	-1.6(5)	9.3(6)	-0.7(5)

P4	12.9(6)	11.4(6)	14.1(6)	0.8(5)	8.3(5)	-0.4(5)
P5	15.1(7)	16.7(7)	20.5(8)	2.0(5)	11.6(6)	1.8(5)
P6	14.4(6)	14.0(6)	16.8(7)	-1.0(5)	10.2(6)	-0.2(5)
P7	14.0(7)	17.5(7)	16.2(7)	1.1(6)	10.2(6)	1.4(6)
P8	16.9(7)	13.7(7)	19.4(7)	1.2(6)	9.3(6)	-2.5(6)
P9	13.8(7)	11.8(6)	16.2(7)	0.5(5)	8.9(6)	1.7(5)
P10	13.3(7)	11.7(6)	15.3(7)	-1.0(5)	9.5(6)	-0.8(5)
P11	15.1(7)	16.8(7)	17.7(7)	1.8(5)	11.1(6)	0.8(5)
P12	14.3(7)	14.0(7)	15.9(7)	-1.8(5)	9.5(6)	-0.4(5)
P13	15.3(7)	12.1(6)	15.5(7)	0.2(5)	9.5(6)	-1.6(5)
P14	14.4(7)	13.2(6)	15.3(7)	0.1(5)	9.7(6)	0.6(5)
P15	12.4(6)	13.2(7)	16.7(7)	-0.6(5)	8.9(6)	-0.3(5)
P16	15.7(7)	14.0(6)	15.9(7)	1.2(5)	10.4(6)	2.5(5)
P17	14.9(7)	9.8(6)	17.1(7)	-0.8(5)	9.0(6)	0.6(5)
P18	13.0(7)	13.7(6)	16.5(7)	2.5(5)	7.9(6)	-1.0(5)
P19	14.7(7)	12.8(6)	18.3(7)	-0.6(5)	11.6(6)	-0.7(5)
P20	14.5(7)	10.9(6)	17.1(7)	-0.1(5)	8.8(6)	1.4(5)
O1	41(3)	36(2)	27(2)	-7.6(17)	24(2)	-0.7(19)
O2	28(2)	22(2)	22(2)	7.9(15)	13.1(17)	3.5(16)
O3	24(2)	17(2)	57(3)	0.7(18)	29(2)	2.0(16)
O4	34(2)	39(3)	24(2)	6.6(18)	15.2(19)	-10.1(19)
O5	19.4(18)	25(2)	28(2)	-11.1(16)	17.9(17)	-5.6(15)
O6	26(2)	15.0(18)	27(2)	-7.4(15)	17.2(17)	-5.9(15)
O7	17.5(19)	23(2)	23(2)	-2.2(15)	9.4(16)	6.1(15)
O8	36(2)	19(2)	27(2)	4.1(16)	21(2)	-0.2(17)
O9	31(2)	19(2)	35(2)	6.8(16)	25.4(19)	6.7(16)
O10	31(2)	28(2)	30(2)	2.3(17)	20(2)	8.1(17)
O11	18(2)	41(2)	26(2)	6.3(18)	11.9(17)	-6.2(17)
O12	12.8(17)	21.3(18)	16.0(17)	1.9(14)	8.3(15)	-1.9(14)
O13	26(2)	18.4(19)	25(2)	-7.8(15)	16.2(18)	-2.6(16)
O14	21(2)	16.1(19)	28(2)	-7.7(15)	14.9(18)	-5.2(15)
O15	19.9(19)	26(2)	20.9(19)	9.7(15)	13.3(17)	1.3(15)
O16	17.6(19)	16.3(19)	29(2)	6.8(15)	15.1(17)	4.9(14)
O17	18.0(19)	19.7(19)	28(2)	-6.2(15)	15.0(17)	-2.7(15)
O18	28(2)	11.8(18)	21(2)	0.7(14)	16.8(18)	4.8(15)
O19	15.3(18)	13.1(18)	25(2)	-2.1(14)	10.7(16)	0.8(14)
O20	31(2)	17(2)	31(2)	8.3(16)	18.1(19)	8.7(16)
O21	23(2)	22(2)	24(2)	1.2(15)	16.4(18)	1.6(15)
O22	17.2(19)	16.0(18)	25(2)	0.7(15)	12.4(17)	-1.1(14)
O23	15.0(18)	19.8(18)	21.9(19)	-2.3(15)	10.6(16)	1.8(14)
O24	20.6(19)	26(2)	21.1(19)	4.7(16)	12.6(17)	6.1(16)
O25	27(2)	26(2)	21(2)	-2.8(16)	13.5(18)	-1.7(16)
O26	34(2)	19(2)	28(2)	-7.5(16)	15.6(19)	-9.7(17)
O27	26(2)	20(2)	31(2)	8.3(16)	11.5(19)	1.6(16)

O28	21.5(19)	19.8(19)	26.8(19)	-2.1(15)	19.3(17)	-4.5(15)
O29	36(2)	31(2)	23(2)	-2.1(17)	17.9(18)	4.0(18)
O30	21(2)	14.5(18)	24(2)	4.7(15)	13.7(17)	7.1(15)
O31	18.8(19)	25(2)	26(2)	5.5(16)	15.8(17)	6.0(15)
O32	25(2)	23(2)	18.2(19)	1.0(15)	13.6(17)	1.9(16)
O33	24.0(19)	14.1(18)	34(2)	0.6(15)	22.5(17)	-1.5(15)
O34	16.6(19)	31(2)	19.0(19)	-1.0(16)	11.0(16)	-5.4(16)
O35	27(2)	26(2)	20(2)	-4.0(15)	15.3(18)	-2.4(16)
O36	21(2)	24(2)	22(2)	2.5(16)	12.7(17)	5.0(16)
O37	14.4(17)	21.0(19)	20.8(18)	-4.8(15)	8.2(15)	-0.9(15)
O38	27(2)	28(2)	28(2)	4.2(17)	18.9(19)	1.7(17)
O39	32(2)	24(2)	26(2)	-4.0(16)	19.9(19)	5.5(17)
O40	18.0(19)	16.7(19)	35(2)	1.7(16)	18.3(17)	1.2(14)
O41	25(2)	16.1(19)	24(2)	-5.5(15)	15.2(18)	-3.6(15)
O42	21(2)	19.7(19)	21(2)	6.2(15)	11.2(17)	3.9(16)
O43	27(2)	15.7(18)	22(2)	1.0(14)	17.2(17)	-3.5(15)
O44	15.3(18)	12.0(17)	33(2)	-1.5(15)	13.5(17)	1.1(14)
O45	36(2)	23(2)	21(2)	0.5(16)	18.6(19)	3.7(17)
O46	12.7(18)	31(2)	21.7(19)	5.8(16)	9.7(16)	-0.5(15)
O47	19.6(19)	13.2(18)	26(2)	-5.3(15)	12.3(17)	-3.5(14)
O48	32(2)	14.1(18)	23.3(19)	-5.6(15)	19.9(17)	-3.1(15)
O49	26(2)	19.0(19)	26(2)	6.3(15)	13.9(17)	2.4(16)
O50	20(2)	16.5(19)	35(2)	7.1(16)	13.1(18)	2.3(15)
O51	28(2)	21(2)	33(2)	-9.6(16)	18.2(19)	-4.1(16)
O52	24(2)	15.9(19)	26(2)	-5.8(15)	15.1(18)	-1.7(16)
O53	19(2)	38(2)	27(2)	11.5(17)	11.8(18)	-5.3(17)
O54	24(2)	19(2)	38(2)	-10.5(17)	23.1(19)	-6.4(16)
O55	15.3(19)	15.3(18)	31(2)	-6.1(15)	11.9(17)	-0.4(14)
O56	35(2)	17(2)	34(2)	8.2(17)	15(2)	7.0(17)
Li1	35(6)	21(5)	20(5)	0(4)	18(4)	3(4)
Li2	42(7)	45(7)	25(5)	15(5)	9(5)	-7(5)
Li3	62(8)	17(5)	27(5)	1(4)	27(5)	-7(5)
Li4	14(4)	12(4)	32(5)	1(3)	13(4)	3(3)
Li5	67(8)	20(5)	23(5)	1(4)	28(5)	-6(5)
Li6	29(5)	13(4)	22(5)	2(3)	16(4)	-3(4)
Li7	20(5)	18(5)	22(5)	2(3)	11(4)	2(4)
Li8	24(5)	21(5)	30(5)	-5(4)	18(4)	-2(4)
Li9	41(6)	21(5)	24(5)	-7(4)	16(5)	0(4)
Li10	29(6)	40(6)	36(6)	-15(5)	10(5)	7(5)
Li11	100(10)	13(5)	36(6)	6(4)	46(7)	7(5)
Li12	27(5)	13(4)	21(5)	-2(3)	13(4)	-6(4)

Table S5. Anisotropic displacement parameters ($Å^2 \times 10^3$) for $Li_4P_6O_{17}$. The anisotropic displacement factor exponent takes the form: $-2\pi^2[h^2a^{*2}U_{11} + 2hka^*b^*U_{12} + \ldots]$.

Atom	U_{11}	U_{22}	U_{33}	U_{23}	U_{13}	U_{12}
P1	4.9(2)	5.1(2)	5.2(2)	-0.23(16)	-0.57(16)	-1.53(16)
P2	5.0(2)	4.8(2)	4.9(2)	0.04(16)	-0.76(16)	-1.17(16)
P3	4.8(2)	5.0(2)	4.9(2)	-0.29(16)	-0.56(16)	-1.14(16)
P4	5.4(2)	4.7(2)	6.2(2)	0.26(16)	-1.68(16)	-1.43(16)
P5	4.9(2)	4.5(2)	5.2(2)	-0.33(16)	-1.10(16)	-1.25(16)
P6	4.8(2)	4.8(2)	5.6(2)	0.03(16)	-0.57(16)	-1.44(16)
O1	10.6(6)	7.9(6)	6.1(6)	-1.4(5)	0.1(5)	-2.5(5)
O2	8.8(6)	7.6(6)	8.9(6)	-0.1(5)	-3.1(5)	-3.2(5)
O3	5.1(6)	6.9(6)	12.1(6)	-2.0(5)	-2.2(5)	-0.6(5)
O4	10.0(6)	5.5(6)	6.7(6)	0.0(5)	-0.1(5)	-3.5(5)
O5	11.5(6)	5.4(6)	4.8(6)	0.8(5)	0.2(5)	-0.7(5)
O6	6.5(6)	9.6(6)	7.9(6)	-2.0(5)	0.1(5)	-0.7(5)
O7	7.1(6)	7.6(6)	6.6(6)	-0.2(5)	1.0(5)	-2.4(5)
O8	6.2(6)	10.2(6)	7.9(6)	-1.9(5)	-1.0(5)	-3.0(5)
O9	9.4(6)	6.8(6)	6.5(6)	-1.8(5)	-0.7(5)	-2.5(5)
O10	10.5(6)	11.7(6)	6.5(6)	0.1(5)	-1.2(5)	-3.6(5)
O11	9.4(6)	6.3(6)	11.4(6)	-0.6(5)	-4.2(5)	-2.4(5)
O12	8.2(6)	4.1(6)	8.2(6)	0.7(5)	-3.3(5)	-1.3(5)
O13	8.1(6)	8.9(6)	9.1(6)	-0.6(5)	-3.1(5)	-2.9(5)
O14	8.2(6)	5.2(6)	6.1(6)	0.0(5)	-0.3(5)	-2.6(5)
O15	5.0(6)	7.3(6)	6.1(6)	-1.2(5)	-0.3(5)	-0.1(5)
O16	10.6(6)	6.0(6)	7.7(6)	-0.7(5)	-2.4(5)	-2.2(5)
O17	6.3(6)	8.3(6)	8.7(6)	0.1(5)	0.4(5)	-1.8(5)
Li1	10.8(15)	13.7(15)	6.9(15)	0.8(12)	-2.1(12)	-3.5(12)
Li2	14.6(16)	10.8(15)	9.2(15)	-0.1(12)	-1.0(12)	-2.3(13)
Li3	11.0(15)	9.5(15)	7.5(14)	-0.1(12)	0.2(12)	-2.8(12)
Li4	10.2(15)	9.8(15)	11.1(15)	-1.2(12)	-0.8(12)	-1.0(12)

Table S6. Bond lengths for $Li_3P_5O_{14}$.

Atom	Atom	Length/Å	Atom	Atom	Length/Å
P1	O1	1.448(4)	P17	O48	1.622(4)
P1	O2	1.475(4)	P17	O49	1.447(4)
P1	O3	1.590(4)	P18	O34	1.610(3)
P1	O33[1]	1.619(3)	P18	O50	1.461(4)
P2	O3	1.548(4)	P18	O51	1.473(4)
P2	O4	1.433(4)	P18	O53	1.614(4)
P2	O5	1.553(4)	P19	O33	1.555(4)
P2	O12	1.568(3)	P19	O53	1.542(4)
P3	O5	1.611(4)	P19	O54	1.453(4)

P3	O6	1.475(4)	P19	O55	1.560(3)
P3	O7	1.610(4)	P20	O39[3]	1.609(4)
P3	O8	1.460(4)	P20	O52	1.471(4)
P4	O12	1.618(3)	P20	O55	1.617(3)
P4	O13	1.472(4)	P20	O56	1.456(4)
P4	O15	1.613(3)	O1	Li3	1.807(10)
P4	O16	1.471(3)	O2	Li4	1.933(10)
P5	O7	1.573(4)	O2	Li9[2]	1.974(10)
P5	O9	1.545(4)	O4	Li2	1.883(10)
P5	O10	1.449(4)	O6	Li1	1.892(11)
P5	O11	1.553(4)	O6	Li3[6]	1.948(10)
P6	O18	1.570(4)	O8	Li2[6]	2.178(13)
P6	O21	1.456(4)	O8	Li5[4]	1.973(10)
P6	O22	1.552(4)	O10	Li8	1.899(9)
P6	O23	1.566(3)	O13	Li7	1.992(10)
P7	O23	1.619(3)	O13	Li8[7]	2.043(10)
P7	O24	1.477(4)	O14	Li2[6]	1.959(11)
P7	O25	1.466(4)	O14	Li5[4]	2.250(13)
P7	O46[2]	1.617(4)	O16	Li6[8]	1.921(9)
P8	O9[3]	1.624(4)	O16	Li12[5]	2.016(10)
P8	O22	1.631(3)	O17	Li4	1.933(9)
P8	O26	1.459(4)	O17	Li5[4]	1.986(10)
P8	O27	1.464(4)	O20	Li3[4]	1.840(11)
P9	O17	1.477(4)	O21	Li8[7]	1.983(9)
P9	O18	1.616(4)	O24	Li1[3]	1.936(10)
P9	O19	1.624(3)	O24	Li10[2]	2.004(11)
P9	O20	1.453(4)	O25	Li5	1.869(10)
P10	O14	1.458(4)	O26	Li6	1.901(9)
P10	O15	1.556(3)	O27	Li7	1.825(9)
P10	O19	1.562(3)	O29	Li11[5]	1.826(11)
P10	O28	1.555(3)	O30	Li7[4]	1.956(10)
P11	O11	1.615(4)	O30	Li8[3]	2.025(10)
P11	O35	1.463(4)	O32	Li9[7]	2.053(10)
P11	O36	1.475(4)	O35	Li12[5]	1.920(10)
P11	O37	1.623(4)	O36	Li2[6]	2.050(11)
P12	O37	1.566(4)	O36	Li6[4]	2.018(10)
P12	O38	1.446(4)	O38	Li10[7]	1.915(11)
P12	O39	1.553(4)	O41	Li4[9]	1.901(9)
P12	O40	1.558(4)	O41	Li9[7]	2.187(11)
P13	O40	1.606(4)	O42	Li1[10]	1.897(10)
P13	O41	1.477(4)	O42	Li3[11]	2.203(12)
P13	O42	1.468(4)	O45	Li9	1.912(10)
P13	O43	1.612(4)	O47	Li6[4]	1.978(10)
P14	O28	1.618(3)	O47	Li12[8]	1.950(9)

P14	O29	1.449(4)	O49	Li11	1.819(10)
P14	O30	1.482(3)	O50	Li4[9]	1.925(9)
P14	O31	1.609(3)	O50	Li5[11]	2.436(13)
P15	O31	1.555(3)	O51	Li1[9]	2.005(10)
P15	O32	1.449(4)	O51	Li10[5]	2.151(13)
P15	O34	1.568(4)	O52	Li7[4]	1.921(10)
P15	O48	1.559(4)	O52	Li11	1.940(10)
P16	O43	1.550(4)	O54	Li10	1.890(11)
P16	O44	1.562(3)	O54	Li11	2.323(15)
P16	O45	1.458(4)	O56	Li12	1.850(9)
P16	O46	1.559(4)			
P17	O44	1.626(4)			
P17	O47	1.477(4)			

Symmetry transformations used to generate equivalent atoms:

[1]1/2+X,1/2+Y,+Z; [2]1/2+X,-1/2+Y,+Z; [3]+X,-1+Y,+Z; [4]+X,1-Y,1/2+Z; [5]+X,1-Y,-1/2+Z; [6]+X,2-Y,1/2+Z; [7]+X,2-Y,-1/2+Z; [8]+X,1+Y,+Z; [9]-1/2+X,3/2-Y,-1/2+Z; [10]-1/2+X,5/2-Y,-1/2+Z; [11]-1/2+X,1/2+Y,+Z

Table S7. Bond lengths for $Li_4P_6O_{17}$.

Atom	Atom	Length/Å	Atom	Atom	Length/Å
P1	O1	1.4748(13)	P6	O16	1.4855(13)
P1	O2	1.4686(13)	P6	O17	1.4662(13)
P1	O3	1.6207(13)	O1	Li2[4]	2.195(3)
P1	O4[1]	1.6295(13)	O1	Li4[5]	1.941(3)
P2	O3	1.5666(13)	O2	Li1[6]	2.017(4)
P2	O4	1.5628(13)	O2	Li2[7]	2.077(3)
P2	O5	1.5657(13)	O6	Li1	1.967(3)
P2	O6	1.4623(13)	O6	Li2[8]	2.313(3)
P3	O5	1.6300(13)	O7	Li3[2]	1.896(3)
P3	O7	1.4799(13)	O7	Li3[6]	1.928(3)
P3	O8	1.5897(13)	O9	Li1[8]	1.983(3)
P3	O9	1.4785(13)	O9	Li2	2.119(3)
P4	O8	1.6039(13)	O10	Li1	1.865(3)
P4	O10	1.4667(13)	O11	Li3	1.900(3)
P4	O11	1.4824(13)	O11	Li4[4]	1.958(3)
P4	O12	1.6358(13)	O13	Li2	2.036(4)
P5	O12	1.5576(13)	O13	Li4[6]	2.433(3)
P5	O13	1.4575(13)	O16	Li3[9]	1.903(3)
P5	O14	1.5782(13)	O16	Li4[10]	2.087(3)
P5	O15	1.5762(13)	O17	Li2[11]	2.078(3)
P6	O14[3]	1.6263(13)	O17	Li4	1.938(3)
P6	O15	1.6131(13)			

Symmetry transformations used to generate equivalent atoms:

^{1}2-X,-Y,1-Z; ^{2}1-X,1-Y,-Z; ^{3}-X,2-Y,-Z; ^{4}1+X,-1+Y,+Z; ^{5}2+X,-1+Y,+Z; ^{6}1+X,+Y,+Z; ^{7}2-X,1-Y,1-Z; ^{8}1-X,1-Y,1-Z; ^{9}-X,1-Y,-Z; ^{10}-1-X,2-Y,-Z; ^{11}-1+X,+Y,+Z

Table S8. Bond angles for $Li_3P_5O_{14}$.

Atom	Atom	Atom	Angle/°	Atom	Atom	Atom	Angle/°
O1	P1	O2	121.0(2)	O34	P18	O53	98.16(19)
O1	P1	O3	111.0(2)	O50	P18	O34	110.1(2)
O1	P1	O33^{1}	110.8(2)	O50	P18	O51	121.7(2)
O2	P1	O3	105.1(2)	O50	P18	O53	106.0(2)
O2	P1	O33^{1}	107.5(2)	O51	P18	O34	109.7(2)
O3	P1	O33^{1}	99.13(19)	O51	P18	O53	108.5(2)
O3	P2	O5	102.5(2)	O33	P19	O55	105.13(19)
O3	P2	O12	100.57(19)	O53	P19	O33	102.3(2)
O4	P2	O3	117.3(2)	O53	P19	O55	105.8(2)
O4	P2	O5	114.9(2)	O54	P19	O33	112.9(2)
O4	P2	O12	115.0(2)	O54	P19	O53	115.4(2)
O5	P2	O12	104.56(19)	O54	P19	O55	114.2(2)
O6	P3	O5	105.1(2)	O39^{2}	P20	O55	97.62(19)
O6	P3	O7	109.8(2)	O52	P20	O39^{2}	105.5(2)
O7	P3	O5	98.38(19)	O52	P20	O55	109.75(19)
O8	P3	O5	111.1(2)	O56	P20	O39^{2}	111.2(2)
O8	P3	O6	121.5(2)	O56	P20	O52	122.9(2)
O8	P3	O7	108.5(2)	O56	P20	O55	107.0(2)
O13	P4	O12	109.3(2)	O6	Li1	O24^{7}	125.4(5)
O13	P4	O15	109.3(2)	O6	Li1	O42^{13}	102.3(5)
O15	P4	O12	98.69(18)	O6	Li1	O51^{14}	106.9(5)
O16	P4	O12	109.2(2)	O24^{7}	Li1	O51^{14}	99.6(4)
O16	P4	O13	121.6(2)	O42^{13}	Li1	O24^{7}	105.2(5)
O16	P4	O15	106.3(2)	O42^{13}	Li1	O51^{14}	118.8(5)
O9	P5	O7	105.0(2)	O4	Li2	O8^{8}	98.6(5)
O9	P5	O11	102.2(2)	O4	Li2	O14^{8}	147.2(6)
O10	P5	O7	113.3(2)	O4	Li2	O36^{8}	101.3(5)
O10	P5	O9	114.7(2)	O14^{8}	Li2	O8^{8}	82.1(4)
O10	P5	O11	117.0(2)	O14^{8}	Li2	O36^{8}	104.3(5)
O11	P5	O7	103.2(2)	O36^{8}	Li2	O8^{8}	126.9(6)
O21	P6	O18	109.5(2)	O1	Li3	O6^{8}	110.3(5)
O21	P6	O22	115.8(2)	O1	Li3	O20^{5}	137.7(6)
O21	P6	O23	117.4(2)	O1	Li3	O42^{3}	102.3(5)
O22	P6	O18	108.8(2)	O6^{8}	Li3	O42^{3}	90.4(4)

O22	P6	O23	101.23(19)	O20^{5}	Li3	O6^{8}	104.8(5)
O23	P6	O18	103.1(2)	O20^{5}	Li3	O42^{3}	100.2(5)
O24	P7	O23	107.1(2)	O2	Li4	O17	122.2(5)
O24	P7	O46^{3}	109.2(2)	O41^{14}	Li4	O2	93.3(4)
O25	P7	O23	110.4(2)	O41^{14}	Li4	O17	117.6(5)
O25	P7	O24	121.6(2)	O41^{14}	Li4	O50^{14}	119.1(5)
O25	P7	O46^{3}	107.7(2)	O50^{14}	Li4	O2	107.4(4)
O46^{3}	P7	O23	98.42(18)	O50^{14}	Li4	O17	98.5(4)
O9^{2}	P8	O22	97.78(19)	O8^{5}	Li5	O14^{5}	80.0(4)
O26	P8	O9^{2}	110.8(2)	O8^{5}	Li5	O17^{5}	131.6(5)
O26	P8	O22	106.6(2)	O8^{5}	Li5	O50^{3}	89.7(4)
O26	P8	O27	124.2(2)	O14^{5}	Li5	O50^{3}	156.9(5)
O27	P8	O9^{2}	105.7(2)	O17^{5}	Li5	O14^{5}	89.5(4)
O27	P8	O22	108.6(2)	O17^{5}	Li5	O50^{3}	82.0(4)
O17	P9	O18	105.9(2)	O25	Li5	O8^{5}	116.6(5)
O17	P9	O19	109.14(19)	O25	Li5	O14^{5}	95.8(5)
O18	P9	O19	98.13(19)	O25	Li5	O17^{5}	111.4(5)
O20	P9	O17	123.4(2)	O25	Li5	O50^{3}	107.3(5)
O20	P9	O18	109.8(2)	O16^{2}	Li6	O36^{5}	111.6(5)
O20	P9	O19	107.6(2)	O16^{2}	Li6	O47^{5}	90.7(4)
O14	P10	O15	114.8(2)	O26	Li6	O16^{2}	119.5(5)
O14	P10	O19	114.7(2)	O26	Li6	O36^{5}	106.9(4)
O14	P10	O28	112.0(2)	O26	Li6	O47^{5}	104.7(5)
O15	P10	O19	105.9(2)	O47^{5}	Li6	O36^{5}	123.8(5)
O28	P10	O15	102.9(2)	O27	Li7	O13	109.2(5)
O28	P10	O19	105.33(19)	O27	Li7	O30^{5}	103.5(5)
O11	P11	O37	99.8(2)	O27	Li7	O52^{5}	112.2(5)
O35	P11	O11	105.9(2)	O30^{5}	Li7	O13	93.2(4)
O35	P11	O36	122.9(2)	O52^{5}	Li7	O13	117.4(5)
O35	P11	O37	110.2(2)	O52^{5}	Li7	O30^{5}	119.1(5)
O36	P11	O11	109.6(2)	O10	Li8	O13^{6}	111.0(4)
O36	P11	O37	106.2(2)	O10	Li8	O21^{6}	121.0(5)
O38	P12	O37	116.3(2)	O10	Li8	O30^{7}	104.2(4)
O38	P12	O39	110.5(2)	O21^{6}	Li8	O13^{6}	111.1(4)
O38	P12	O40	115.0(2)	O21^{6}	Li8	O30^{7}	115.4(4)
O39	P12	O37	104.6(2)	O30^{7}	Li8	O13^{6}	89.7(4)
O39	P12	O40	107.4(2)	O2^{12}	Li9	O32^{6}	101.7(4)
O40	P12	O37	102.08(19)	O2^{12}	Li9	O41^{6}	84.0(4)
O40	P13	O43	98.7(2)	O32^{6}	Li9	O41^{6}	102.4(4)
O41	P13	O40	104.9(2)	O45	Li9	O2^{12}	103.2(4)

O41	P13	O43	110.2(2)	O45	Li9	$O32^{6}$	143.8(6)
O42	P13	O40	110.6(2)	O45	Li9	$O41^{6}$	105.9(5)
O42	P13	O41	123.4(2)	$O24^{12}$	Li10	$O51^{4}$	92.8(5)
O42	P13	O43	106.3(2)	$O38^{6}$	Li10	$O24^{12}$	111.4(5)
O29	P14	O28	111.0(2)	$O38^{6}$	Li10	$O51^{4}$	104.7(5)
O29	P14	O30	122.1(2)	O54	Li10	$O24^{12}$	111.7(5)
O29	P14	O31	110.9(2)	O54	Li10	$O38^{6}$	120.2(6)
O30	P14	O28	106.7(2)	O54	Li10	$O51^{4}$	112.4(6)
O30	P14	O31	106.1(2)	$O29^{4}$	Li11	O52	104.5(5)
O31	P14	O28	97.19(18)	$O29^{4}$	Li11	O54	116.8(6)
O31	P15	O34	100.66(19)	O49	Li11	$O29^{4}$	129.3(7)
O31	P15	O48	104.8(2)	O49	Li11	O52	117.8(6)
O32	P15	O31	118.2(2)	O49	Li11	O54	91.3(5)
O32	P15	O34	115.2(2)	O52	Li11	O54	89.7(5)
O32	P15	O48	111.8(2)	$O35^{4}$	Li12	$O16^{4}$	113.2(4)
O48	P15	O34	104.6(2)	$O35^{4}$	Li12	$O47^{2}$	122.7(5)
O43	P16	O44	108.4(2)	$O47^{2}$	Li12	$O16^{4}$	88.7(4)
O43	P16	O46	102.0(2)	O56	Li12	$O16^{4}$	101.1(4)
O45	P16	O43	111.7(2)	O56	Li12	$O35^{4}$	121.2(5)
O45	P16	O44	114.9(2)	O56	Li12	$O47^{2}$	103.5(4)
O45	P16	O46	117.6(2)				
O46	P16	O44	100.8(2)				
O47	P17	O44	108.81(19)				
O47	P17	O48	104.8(2)				
O48	P17	O44	98.57(19)				
O49	P17	O44	108.4(2)				
O49	P17	O47	123.1(2)				
O49	P17	O48	110.5(2)				

Symmetry transformations used to generate equivalent atoms:

^{1}1/2+X,1/2+Y,+Z; ^{2}+X,-1+Y,+Z; ^{3}1/2+X,-1/2+Y,+Z; ^{4}+X,1-Y,1/2+Z; ^{5}+X,1-Y,-1/2+Z; ^{6}+X,2-Y,1/2+Z; ^{7}+X,1+Y,+Z; ^{8}+X,2-Y,-1/2+Z; ^{9}-1/2+X,-1/2+Y,+Z; ^{10}-1/2+X,3/2-Y,-1/2+Z; ^{11}-1/2+X,5/2-Y,-1/2+Z; ^{12}-1/2+X,1/2+Y,+Z; ^{13}1/2+X,5/2-Y,1/2+Z; ^{14}1/2+X,3/2-Y,1/2+Z; ^{15}+X,-Y,1/2+Z

Table S9. Bond angles for $Li_4P_6O_{17}$.

Atom	Atom	Atom	Angle/°	Atom	Atom	Atom	Angle/°
O1	P1	O3	110.02(7)	O6	Li1	$O2^{11}$	90.50(14)
O1	P1	$O4^{1}$	108.26(7)	O6	Li1	$O9^{8}$	91.57(14)
O2	P1	O1	122.97(8)	$O9^{8}$	Li1	$O2^{11}$	88.92(14)
O2	P1	O3	106.50(7)	O10	Li1	$O2^{11}$	106.41(16)

O2	P1	O4[1]	107.99(7)	O10	Li1	O6	107.59(16)
O3	P1	O4[1]	98.29(7)	O10	Li1	O9[8]	155.0(2)
O4	P2	O3	107.82(7)	O1[12]	Li2	O6[8]	86.95(12)
O4	P2	O5	99.68(7)	O2[7]	Li2	O1[12]	97.35(14)
O5	P2	O3	105.87(7)	O2[7]	Li2	O6[8]	80.08(12)
O6	P2	O3	110.39(7)	O2[7]	Li2	O9	83.79(13)
O6	P2	O4	116.73(7)	O2[7]	Li2	O17[6]	97.35(15)
O6	P2	O5	115.33(7)	O9	Li2	O1[12]	165.81(18)
O7	P3	O5	105.16(7)	O9	Li2	O6[8]	79.29(12)
O7	P3	O8	105.93(7)	O13	Li2	O1[12]	80.87(12)
O8	P3	O5	101.39(7)	O13	Li2	O2[7]	176.08(19)
O9	P3	O5	109.22(7)	O13	Li2	O6[8]	96.31(14)
O9	P3	O7	120.41(7)	O13	Li2	O9	97.07(15)
O9	P3	O8	112.85(7)	O13	Li2	O17[6]	85.99(13)
O8	P4	O12	99.36(7)	O17[6]	Li2	O1[12]	84.72(13)
O10	P4	O8	112.25(7)	O17[6]	Li2	O6[8]	170.89(18)
O10	P4	O11	121.17(8)	O17[6]	Li2	O9	109.23(15)
O10	P4	O12	110.07(7)	O7[2]	Li3	O7[11]	95.67(15)
O11	P4	O8	107.45(7)	O7[2]	Li3	O11	116.84(17)
O11	P4	O12	104.07(7)	O7[2]	Li3	O16[9]	116.73(17)
O12	P5	O14	101.27(7)	O11	Li3	O7[11]	107.39(16)
O12	P5	O15	105.14(7)	O11	Li3	O16[9]	94.02(15)
O13	P5	O12	115.92(7)	O16[9]	Li3	O7[11]	127.42(17)
O13	P5	O14	115.31(7)	O1[13]	Li4	O11[12]	107.50(16)
O13	P5	O15	112.48(7)	O1[13]	Li4	O13[11]	76.89(12)
O15	P5	O14	105.42(7)	O1[13]	Li4	O16[10]	136.79(18)
O15	P6	O14[3]	100.31(7)	O11[12]	Li4	O13[11]	168.29(17)
O16	P6	O14[3]	107.43(7)	O11[12]	Li4	O16[10]	86.80(14)
O16	P6	O15	109.93(7)	O16[10]	Li4	O13[11]	82.86(12)
O17	P6	O14[3]	108.07(7)	O17	Li4	O1[13]	95.89(15)
O17	P6	O15	106.77(7)	O17	Li4	O11[12]	110.85(16)
O17	P6	O16	122.16(8)	O17	Li4	O13[11]	78.97(12)
				O17	Li4	O16[10]	117.25(16)

Symmetry transformations used to generate equivalent atoms:

[1]2-X,-Y,1-Z; [2]1-X,1-Y,-Z; [3]-X,2-Y,-Z; [4]1+X,-1+Y,+Z; [5]2+X,-1+Y,+Z; [6]1+X,+Y,+Z; [7]2-X,1-Y,1-Z; [8]1-X,1-Y,1-Z;

[9]-X,1-Y,-Z; [10]-1-X,2-Y,-Z; [11]-1+X,+Y,+Z; [12]-1+X,1+Y,+Z; [13]-2+X,1+Y,+Z

Table S10. Comparison of structural symmetry, Li polyhedral arrangement, Li–Li distances, ionic conductivities, and activation energy for polycrystalline materials in Li–P–O phase field.

Compound	Space group	V/Z / Å^3	Li polyhedral arrangement	Minimum Li–Li distance / Å	Ionic conductivity at 303 K / S cm^{-1}	Activation energy / eV	Ref.
β-Li_3PO_4	*Pmn*2$_1$ (31)	77.75	3D network composed of corner shared LiO_4^{7-}	2.871	6.12×10^{-20}	1.43	2
γ-Li_3PO_4	*Pnma* (62)	78.83	3D network composed of corner/edge shared LiO_4^{7-} and LiO_5^{9-}	2.679	1.02×10^{-19}	1.40	2
					8.11×10^{-19}	1.31	3
					9.32×10^{-18}	1.24	4
					4.76×10^{-16} (*a* axis)<br>3.66×10^{-17} (*b* axis)<br>3.60×10^{-16} (*c* axis)	1.14 (*a* axis)<br>1.23 (*b* axis)<br>1.14 (*c* axis)	5
c-Li_3PO_4	$Fm\bar{3}m$ (225)	96.45	3D network composed of face shared LiO_4^{7-} and LiO_6^{11-}	3.152	6.94×10^{-8}	0.51	2
$LiPO_3$	*Pc* (7)	57.47	2D layer composed of corner/edge shared LiO_4^{7-}	2.658	–	–	6
$LiPO_3$	*P*2/*c* (13)	57.44	2D layer composed of corner/edge shared LiO_4^{7-}	2.616	7.42×10^{-19}	1.4	7-8
$LiPO_3$	*P*2$_1$/*c* (14)	59.53	2D layer composed of corner/edge shared LiO_4^{7-} and LiO_5^{9-}	2.917	–	–	9
$Li_4P_2O_7$-tric	$P\bar{1}$ (2)	142.35	3D network composed of corner/edge shared LiO_4^{7-}	2.645	1.39×10^{-18}	1.62	10-11
$Li_4P_2O_7$-pseudomono	$P\bar{1}$ (2)		–	–	–	–	12
$Li_4P_2O_7$ -mono-RT	*P*2$_1$/*c* (14)	142.53	3D network composed of corner/edge shared LiO_4^{7-}	2.630	–	–	13
$Li_4P_2O_7$-mono-HT	*P*2$_1$/*c* (14)	148	3D network composed of corner/edge shared LiO_3^{5-} and LiO_4^{7-}	2.151	5.51×10^{-11}	0.83	11
					4.14×10^{-17}	0.95	14
$Li_4P_2O_7$-trig	*P*3$_2$12 (153)	–	–	–	–	–	12
$Li_3P_5O_{14}$	*Cc* (9)	272.66	two types finite $Li_6O_{16}^{26-}$ chain composed of corner/edge shared LiO_4^{7-} and LiO_5^{9-}	2.582	8.5(5)×10^{-7}	0.43(7)	This work
$Li_4P_6O_{17}$	$P\bar{1}$ (2)	329.95	finite $Li_8O_{20}^{32-}$ chain composed of edge/face shared LiO_4^{7-}, LiO_5^{9-}, and LiO_6^{11-}	2.568	–	–	This work

Table S11. Summary of the refinement parameters of $Li_3P_5O_{14}$ against PXRD data.

	Pawley fit	Rietveld fit
Empirical formula	$Li_3P_5O_{14}$	
Formula weight	399.67	
Temperature/K	293(2)	
Wavelength (Å)	1.540596	
Crystal system, Space group	monoclinic, *Cc*	
a/Å	33.4045(8)	33.3976(6)
b/Å	11.0123(4)	11.0087(2)
c/Å	15.1021(7)	15.0980(3)
β/°	128.059(2)	128.060(1)
R_p	3.05%	3.54%
R_{wp}	4.04%	4.72%
R_{exp}	3.16%	3.58%
χ^2	1.64	1.74

Table S12. Impedance fitted parameters of $Li_3P_5O_{14}$ at 303 K.

Element	Value
R_b / MΩ cm	1.06(4)
Q_b	$4.88(6) \times 10^{-10}$
n_b	0.667(3)
R_{gb} / MΩ cm	0.30(1)
Q_b	$1.00(5) \times 10^{-10}$
n_{gb}	0.759(6)

Both AC and DC interfacial resistivities are calculated based on the thickness and diameter of the pellets (Table S13), following a reported procedure.[15] For the AC data, the total resistivity of the Li|$Li_3P_5O_{14}$|Li cell (sum of the resistivity of $Li_3P_5O_{14}$ solid electrolyte, and resistivity of two interfaces between $Li_3P_5O_{14}$|Li) and the resistivity of the $Li_3P_5O_{14}$ solid electrolyte were determined from the room-temperature AC impedance data of the Li|$Li_3P_5O_{14}$|Li cell (Figure S14) and the Au|$Li_3P_5O_{14}$|Au cell (Figure 11a), respectively. For the DC data, the total resistivity of the Li|$Li_3P_5O_{14}$|Li cell and the resistivity of the $Li_3P_5O_{14}$ solid electrolyte were determined from the DC plating/stripping data of the Li|$Li_3P_5O_{14}$|Li cell (Figure 11g) and the DC polarization test of the Au|$Li_3P_5O_{14}$|Au cell (Figure 11d), respectively.

Table S13. Interfacial resistivity at room temperature calculated from the AC and DC measurements for the Au|$Li_3P_5O_{14}$|Au and Li|$Li_3P_5O_{14}$|Li cells.

	Li|$Li_3P_5O_{14}$|Li cell	Au|$Li_3P_5O_{14}$|Au cell	Interfacial resistivity / MΩ cm
	Total resistivity / MΩ cm	Resistivity of $Li_3P_5O_{14}$ / MΩ cm	
AC impedance	6.47	1.36	2.55
DC measurement	52	33	9.5

REFERENCES

1. Perdew, J. P.; Burke, K.; Ernzerhof, M., Generalized Gradient Approximation Made Simple [Phys. Rev. Lett. 77, 3865 (1996)]. *Phys. Rev. Lett.* **1997,** *78* (7), 1396-1396.

2. Zhu, B.; Mellander, B.-E.; Chen, J., Cubic Alkali Orthophosphates with High Ionic Conductivity. *Mater. Res. Bull.* **1993,** *28* (4), 321-328.

3. Hu, Y. W.; Raistrick, I. D.; Huggins, R. A., Ionic Conductivity of Lithium Orthosilicate—Lithium Phosphate Solid Solutions. *J. Electrochem. Soc.* **1977,** *124* (8), 1240-1242.

4. Wang, B.; Chakoumakos, B. C.; Sales, B. C.; Kwak, B. S.; Bates, J. B., Synthesis, Crystal Structure, and Ionic Conductivity of a Polycrystalline Lithium Phosphorus Oxynitride with the γ-Li_3PO_4 Structure. *J. Solid State Chem.* **1995,** *115* (2), 313-323.

5. Ivanov-Shitz, A. K.; Kireev, V. V.; Mel'nikov, O. K.; Demianets, L. N., Growth and Ionic Conductivity of *γ*-Li_3PO_4. *Crystallogr. Rep.* **2001,** *46* (5), 864-867.

6. Guitel, J.-C.; Tordjman, I., Structure Cristalline de Polyphosphate de Lithium $LiPO_3$. *Acta Cryst.* **1976,** *B32* (11), 2960-2966.

7. Murashova, E. V.; Chudinova, N. N., Synthesis and Crystal Structures of Lithium Polyphosphates, $LiPO_3$, $Li_4H(PO_3)_5$, and $LiMn(PO_3)_3$. *Crystallogr. Rep.* **2001,** *46* (6), 942-947.

8. Money, B. K.; Hariharan, K., Lithium Ion Conduction in Lithium Metaphosphate Based Systems. *Appl. Phys. A* **2007,** *88* (4), 647-652.

9. Ben-Chaabane, T.; Smiri-Dogguy, L.; Laligant, Y.; Le Bail, A., $Li_6P_6O_{18}$: X-ray Powder Structure Determination of Lithium Cyclohexaphosphate. *Eur. J. Solid State Inorg. Chem.* **1998,** *35* (3), 255-264.

10. Daidouh, A.; Veiga, M. L.; Pico, C.; Martinez-Ripoll, M., A New Polymorph of $Li_4P_2O_7$. *Acta. Crystallogr. C.* **1997,** *53* (2), 167-169.

11. Voronin, V. I.; Sherstobitova, E. A.; Blatov, V. A.; Shekhtman, G. S., Lithium-Cation Conductivity and Crystal Structure of Lithium Diphosphate. *J. Solid State Chem.* **2014,** *211*, 170-175.

12. Raguž, B.; Wittich, K.; Glaum, R., Two New, Metastable Polymorphs of Lithium Pyrophosphate $Li_4P_2O_7$. *Eur. J. Inorg. Chem.* **2019,** *2019* (11-12), 1688-1696.

13. Yakubovich, O. V.; Mel'nikov, O. K., The Crystal Structure of $Li_4[P_2O_7]$. *Crystallogr. Rep.* **1994,** *39*, 737-742.

14. Zaafouri, A.; Megdiche, M.; Gargouri, M., Studies of Electric, Dielectric, and Conduction Mechanism by OLPT Model of $Li_4P_2O_7$. *Ionics* **2015,** *21* (7), 1867-1879.

15. Rosero-Navarro, N. C.; Kajiura, R.; Jalem, R.; Tateyama, Y.; Miura, A.; Tadanaga, K., Significant Reduction

in the Interfacial Resistance of Garnet-Type Solid Electrolyte and Lithium Metal by a Thick Amorphous Lithium Silicate Layer. *ACS Appl. Energy Mater.* **2020,** *3* (6), 5533-5541.
